# Adolescent Expectations of Early Death Predict Adult Risk Behaviors

**DOI:** 10.1371/journal.pone.0041905

**Published:** 2012-08-01

**Authors:** Quynh C. Nguyen, Andres Villaveces, Stephen W. Marshall, Jon M. Hussey, Carolyn T. Halpern, Charles Poole

**Affiliations:** 1 Department of Epidemiology, UNC Gillings School of Global Public Health, University of North Carolina at Chapel Hill, Chapel Hill, North Carolina, United States of America; 2 UNC Injury Prevention Research Center, University of North Carolina at Chapel Hill, Chapel Hill, North Carolina, United States of America; 3 Department of Maternal and Child Health, UNC Gillings School of Global Public Health, Chapel Hill, North Carolina, United States of America; 4 Carolina Population Center, University of North Carolina at Chapel Hill, Chapel Hill, North Carolina, United States of America; University of Western Brittany, France

## Abstract

Only a handful of public health studies have investigated expectations of early death among adolescents. Associations have been found between these expectations and risk behaviors in adolescence. However, these beliefs may not only predict worse adolescent outcomes, but worse trajectories in health with ties to negative outcomes that endure into young adulthood. The objectives of this study were to investigate perceived chances of living to age 35 (Perceived Survival Expectations, PSE) as a predictor of suicidal ideation, suicide attempt and substance use in young adulthood. We examined the predictive capacity of PSE on future suicidal ideation/attempt after accounting for sociodemographics, depressive symptoms, and history of suicide among family and friends to more fully assess its unique contribution to suicide risk. We investigated the influence of PSE on legal and illegal substance use and varying levels of substance use. We utilized the National Longitudinal Study of Adolescent Health (Add Health) initiated in 1994–95 among 20,745 adolescents in grades 7–12 with follow-up interviews in 1996 (Wave II), 2001–02 (Wave III) and 2008 (Wave IV; ages 24–32). Compared to those who were almost certain of living to age 35, perceiving a 50–50 or less chance of living to age 35 at Waves I or III predicted suicide attempt and ideation as well as regular substance use (i.e., exceeding daily limits for moderate drinking; smoking ≥ a pack/day; and using illicit substances other than marijuana at least weekly) at Wave IV. Associations between PSE and detrimental adult outcomes were particularly strong for those reporting persistently low PSE at both Waves I and III. Low PSE at Wave I or Wave III was also related to a doubling and tripling, respectively, of death rates in young adulthood. Long-term and wide-ranging ties between PSE and detrimental outcomes suggest these expectations may contribute to identifying at-risk youth.

## Introduction

Perceptions of immortality and invincibility have been offered as explanations for heightened risk-taking among youth [1]. However, fatalism and perceived vulnerability may also encourage greater recklessness. Cross-sectional studies linked adolescent expectations of early death to risk behaviors like suicide ideation/act, impulsive sensation-seeking, drinking and driving, and selling drugs [2], [3]. Studies utilizing data from the National Longitudinal Study of Adolescent Health (Add Health) identified low perceived chances of living to age 35 (which we term Perceived Survival Expectations, PSE) as a predictor of future suicide attempt, fight-related injury, unsafe sexual activity, police arrest, human immunodeficiency virus (HIV) diagnosis [4]–and most recently to cigarette smoking [5]. The importance of low PSE is underscored by their prevalence among youth. One in seven adolescents at Wave I of Add Health reported perceiving a 50–50 chance or less of living to age 35 [4].

Low PSE may reflect an overall tendency to view the future pessimistically, fatalistically and with resignation. Hopelessness has been linked with violence involvement, substance use and early sexual activity among adolescents [6]. As described above, analogous associations have been found for low PSE [4]. Hopelessness may impair an individual’s abilities to define problems and to formulate and implement their solutions [7]. Recently, low PSE were linked to avoidance of problems and acting without considering consequences [8]. Essential components of hopelessness include the expectation that desired events will not occur, negative events will occur, and nothing can be done to change the course of these events. Beck’s original 20-item true/false hopelessness scale was composed of items such as “My future seems dark to me,” and “I might as well give up because I can’t make things better for myself” [9]. There are current movements to incorporate considerations of survival expectations more directly into hopelessness theory. For instance, Bolland added the following item to his hopelessness scale: “I don’t expect to live a very long life” [6].

### Suicide and Substance Use among Young Adults

Among those 25–34 years old, suicide represents the second leading cause of death (12.3 suicide deaths per 100,000) [10]. One-third of suicide attempts result in injuries that require medical attention [11]. Suicidal behavior among youths is strongly related to psychiatric disorders including major depressive disorder, antisocial behaviors, and substance use disorder [12]. Hopelessness is also tied to suicidal behavior [13], [14]. Other key risk factors for suicide include family history of suicide and psychopathology, stressful life events such as child abuse, violence exposure, and social/economic disadvantage [12], [15]. Structural factors related to suicide include neighborhood poverty/economic deprivation [16]. Protective factors include family cohesion (i.e., mutual involvement, shared interests, emotional support) and religiosity [17]. Suicide deaths are more prevalent among men but women attempt suicide more often [18].

Additionally, substance use has substantial health costs. Cigarette smoking is the largest individual cause of preventable deaths and morbidity across the world [19]. Alcohol accounts for approximately 3% of global deaths and 4% of disability-adjusted life years (DALYs) [20]. Illicit substance use is connected with excess mortality, fatal and non-fatal injuries, motor-vehicle accidents, violence and psychiatric disorders [7]. Substance use peaks in young adulthood, reaching 72% by age 27. Daily drinking, binge drinking and daily drunkenness are at their maxima in young adulthood [21].

Identified risk factors for substance use are extensive and include regulations and norms favorable to drug use, availability of drugs, severe economic deprivation, psychiatric disorders, family history of substance use, family conflict, low family attachment, low school commitment, early peer rejection, social pressure, alienation and rebelliousness [22]. Men have higher rates of substance use and problem use than women [23], [24]. Personality traits hypothesized to be associated with substance use include hopelessness, impulsivity, anxiety sensitivity, and sensation-seeking [7].

### PSE as a Predictor of Young Adult Risk Behaviors

Previously, low PSE has been connected to a variety of adverse health outcomes including fight-related injury, diagnosis of HIV/AIDS, and police arrests [4]. In this study, we investigate expectations of an early death as a predictor of young adult suicidal behavior and substance use–two categories of risk behaviors that exert large health burdens on this age group. We hypothesize that PSE increases the likelihood of both types of behaviors. People who perceive a severely bleak or limited future may respond to challenges with fatalism and less persistence. They may set fewer goals, seek less guidance, and develop or attempt fewer solutions to their problems [25]. Low PSE in adolescence may increase the risk for suicide later in life through the development of cognitive schemas in which negative events are seen as inevitable and desired events are seen as unattainable. Individuals with low PSE may be more apt to believe that their problems are insoluble and to view suicide as the only recourse. It is also important to assess whether such associations persist even after accounting for depressive symptoms, religiosity, and history of suicide among family and friends along with other potentially confounding factors. Additionally, perceptions of a bleak future may encourage substance use by promoting a disregard of future consequences or encourage substance use as a means to cope. People who perceive poor life chances may act more recklessly [2], [3], [4], [8].

Recently, McDade et al. using Add Health data connected lower PSE to higher daily number of cigarettes among current smokers [5]. Another Add Health study found that adolescents who reported low PSE at Waves I and II were more likely to report past-year illicit substance use compared to those who had high PSE at both waves, although effect measures bordered statistical significance [8]. A third study found Wave I PSE was unrelated to past-year illicit substance use at Waves II and III [4]. Nonetheless, marijuana, the most common illicit substance, was not distinguished from other illicit drugs. Approximately three-quarters of illicit drug users report marijuana use [26]. The health effects of marijuana are also generally more benign than those of other illicit drugs [27]. In addition, these studies did not examine varying levels of use and they did not investigate alcohol use. Previous studies have only examined past-year illicit substance use.

### Study Aims and Hypotheses

The aim of this study was to examine associations between PSE and future suicidal ideation, suicide attempts, and substance use in young adulthood after accounting for important confounding factors that relate to low PSE and to the detrimental outcomes under investigation. This study utilized data from Add Health to test two hypotheses:

1. Low PSE at Waves I and III will be associated with increased suicidal ideation and suicide attempt at Wave IV.
2. Low PSE at Waves I and III will be associated with increased cigarette smoking, alcohol use, marijuana use and other illicit substance use at Wave IV.

The efficiency of PSE cannot be over emphasized. PSE was assessed with one survey question pertaining to respondents’ perceived chances of living to age 35, and Wave I occurred 13–14 years prior to Wave IV. If PSE is predictive of detrimental outcomes at Wave IV even after adjusting for important individual and contextual confounding factors, it merits consideration as a tool to identify at-risk youth.

## Methods

### Study Population

This study utilized data from Add Health, a nationally representative field study of 20,745 U.S. adolescents in grades 7 through 12 during the 1994–1995 school year. The Wave I response rate was 79% (mean age 16) [28]. Three in-home follow-up interviews of the cohort have been completed: Wave II in 1996 (88% of the eligible cohort at Wave I; mean age 16), Wave III in 2001–2002 (77%; mean age 22), and Wave IV in 2008 (80%; mean age 28). By design, high school seniors at Wave I were not re-interviewed at Wave II, but were included in Waves III and IV. At Wave IV, 15,701 respondents aged 24 to 32 years were interviewed. The analytic sample was restricted to respondents with non-missing survey weights (N = 14,800 with Wave IV cross-sectional weights and N = 12,288 with valid longitudinal weights for analyses that use data from Waves I, III, IV).

### Variable Coding/definitions

Variable definitions can be found in the Supplemental Materials (Table S1). The main predictor of interest is *Perceived Survival Expectations (PSE)*. Respondents were asked at Waves I, II and III, “What are your chances of living to age 35?” Possible responses were: “almost no chance;” “some chance, but probably not;” “a 50–50 chance;” “a good chance” and “almost certain.” Consistent with previous literature, we collapsed PSE equals “50–50 chance” with lower categories [4]. We also assessed whether more moderate departures from certainty of living to age 35 (i.e., reporting “a good chance”) were also related to adverse outcomes.

#### Wave IV outcomes

Outcomes under examination included suicidal ideation and attempt as well as substance use at Wave IV. Both *suicidal ideation* (assessed via the question, “During the past 12 months, have you ever seriously thought about committing suicide?”) and *suicide attempt* (assessed via the question, “During the past 12 months, how many times have you actually attempted suicide?”) were coded dichotomously due to low prevalences of both behaviors. All respondents were asked about the number of suicide attempts regardless of their response to the question on suicidal ideation. To investigate whether PSE is differentially related to divergent levels of substance use, we coded substance use with a series of polytomous variables–with the exception of *exceeding recommended daily limits for moderate drinking* which was defined as two or more drinks per day for women and three or more drinks per day for men [29]. *Cigarette smoking* had the following levels: A) none, B) less than daily smoking, C) daily smoking but less than a pack a day, and D) at least a pack a day [30]. *Binge drinking* was defined as 5/4 [men/women] or more drinks in a row [31]. We also investigated *marijuana use* and *use of illicit substances other than marijuana*. Frequency of the latter three outcomes was examined with the following levels: none, monthly or less, 2–3 days/month, at least weekly. This study contributes to the literature by exploring PSE as a predictor of a variety of substance use behaviors (i.e., cigarette smoking, binge drinking, heavy daily drinking, marijuana use, and other illicit substance use)–and at varying levels of use from occasional to regular use.

#### Covariates

The primary aim of this study is to study is to assess PSE as a predictor of suicidal ideation, suicidal attempt, and substance use in young adulthood, independent of important confounding factors. The following covariates were selected a priori based upon the literature demonstrating their relationship to lower PSE and greater engagement in risk behaviors [4], [5], [8], [32]: age (years), sex, foreign-birth, race/ethnicity, parental education, family structure, childhood physical maltreatment, childhood sexual abuse, 12-month family history of suicide, 12-month history of suicide among friends, substance use, self-rated health, religiosity, depressive symptoms and parental attachment/support (Table S1). Indices were constructed for depressive symptoms, religiosity and parental attachment/support. For each index, scores were calculated by averaging across items composing the index. We required valid responses for two-thirds of questions composing the indices. Internal consistency of items composing the indices was satisfactory with Cronbach’s alphas approximately 0.70 or greater. For all variables, higher values indicate greater risk.

### Statistical Analyses

We first calculated prevalence estimates and 95% confidence intervals for PSE, all covariates, and Wave IV outcomes. We then evaluated associations between PSE and Wave IV outcomes. In assessing PSE as a marker of negative health outcomes, we examined both the predictive capacities of PSE measured at Wave I when respondents were in grades 7–12^th^ and PSE measured at Wave III when respondents were 18–26 years old. Adults have more developed risk perceptions compared to adolescents who overestimate the probability of negative events [33]. Thus, low PSE at Wave III may be less subject to crude risk perceptions (compared to Wave I PSE) and may signal severe levels of hopelessness. We also examined the outcomes of respondents who reported persistently low PSE at Waves I and III in order to assess whether this group is particularly vulnerable to detrimental outcomes in young adulthood.

We investigated the potential effects of Wave I PSE on Wave IV suicidal ideation and attempt (Study Aim 1) using log-binomial regression models to calculate relative risks and including all aforementioned covariates. We investigated the relationship between Wave III PSE and Wave IV suicidal behavior controlling for the full set of covariates with Wave III values where available (i.e., family history of suicide, history of suicide among friends, block group poverty, depressive symptoms, religiosity, parental attachment/support, substance use and self-rated health).

Secondly, we investigated the potential impact of Wave I and Wave III PSE on Wave IV substance use (Study Aim 2). For the dichotomous outcome variable (i.e., exceeding daily limits for moderate drinking), we utilized log-binomial regression models to estimate relative risks. For polytomous outcome variables, we utilized multinomial logistic regression because the Brant test showed violations of the proportional odds assumption [34]. Models controlled for all-above specified covariates that were included in models evaluating the relationship between PSE and suicide with the exception of self-reported history of suicide among family and friends–which had negligible impact on effect sizes but would have reduced the analytic sample size (results not shown). In order to investigate whether PSE has an enduring effect on substance use in young adulthood beyond its effects on adolescent substance use [4], we controlled for previous substance use and compared with effect estimates without this adjustment.

We tested the statistical significance of sex-by-PSE interactions in order to evaluate whether the effects of PSE differed for males and females. Across all examined outcomes, these interactions were non-significant at the 0.05 level, and thus not included in final models. Among respondents with valid survey weights, missing data for PSE and all Wave IV outcomes was <1%. Missing data for covariates were also low (generally <2%). Wave III parental attachment/support had the highest rate of missingness (5%). The exclusion of individuals with missing data on PSE, Wave IV outcomes, and covariates resulted in the exclusion of 6–13% of respondents. All summary statistics were produced using STATA®/SE 10 (StataCorp LP, College Station, TX) and weighted to be representative of U.S. adolescents in grades 7–12 in the 1994–95 school year.

### Ethics

This analysis was exempt from further review by the Public Health-Nursing IRB at the University of North Carolina at Chapel Hill.

## Results

### Descriptive Statistics

At Wave I, 14% (95% CI: 13, 16) of adolescents in grades 7–12 reported low PSE (i.e., ≤50% chance of living to age 35) (Table 1). However at Wave III, when all respondents were 18 years and older, the proportion reporting low PSE reduced to 7% (95% CI: 7, 8). Approximately 2.3% (95% CI: 1.9, 2.9) of respondents reported low PSE At Wave I and III (results not shown). At Wave I, the Add Health population was approximately balanced between males and females. Most respondents were white (65%) with large populations of blacks (15%) and Hispanics (11%). About 6% (95% CI: 5, 9) were foreign-born (Table 1). Due to space limitations, we present descriptive statistics for an abbreviated list of covariates in Table 1. Descriptive statistics on all covariates are displayed in Table S2.

**Table 1:** Descriptive Statistics, Add Health.

	Wave I (1994–95)		Wave II (1996)							Wave III (2001–02)						Wave IV (2008)		
	n	% (95% CI) or Mean (SD)^a^	n		% (95% CI) or Mean (SD)^a^					n				% (95% CI) or Mean (SD)^a^		n	% (95% CI) or Mean (SD)^a^	
**Perceived survival expectations**																		
Almost certain	10250	57 (55, 59)	7016	54 (52, 56)					10309				74 (72, 75)					
A good chance	5775	29 (28, 30)	4336	31 (29, 32)					2817				19 (18, 20)					
A 50–50 chance	2065	11 (10, 12)	1670	12 (11, 13)					1020				6.8 (6.1, 7.6)					
Some chance but probably not	439	2 (2, 3)	325	2 (2, 3)					63				0.4 (0.3, 0.5)					
Almost no chance	257	1 (1, 2)	169	1 (1, 2)					30				0.2 (0.1, 0.3)					
**Covariates**																		
Age (years)	18919	16 (2)													14800			28 (2)
Male	9288	51 (50, 52)													6930			51 (49, 52)
*Race*																		
White, non-Hispanic	9608	65 (59, 70)													7849			66 (60, 71)
Black, non-Hispanic	3790	15 (11, 19)													2975			15 (12, 20)
Hispanic	2993	11 (8, 15)													2168			11 (8, 15)
Asian, non-Hispanic	1247	3 (2, 5)													836			3 (2, 5)
Other, non-Hispanic	270	1 (1, 2)													182			1 (1,2)
Multiracial	936	4 (3, 5)													729			4 (3, 5)
Foreign-born	1746	6 (5, 9)													1129			5 (4, 7)
*12-mos family history of suicide*																		
No suicide attempt	17862	95 (95, 96)					13517					97 (97, 98)						
Suicide attempt	658	4 (3, 4)					290					2 (2, 2)						
Suicide attempt resulted in death	170	1 (1, 1)					100					1 (1, 1)						
*12-mos history of suicide among friends*																		
No suicide attempt	15429	82 (81, 83)				12963					93 (92, 93)							
Suicide attempt	2690	15 (14, 16)				601					4 (4, 5)							
Suicide attempt resulted in death	542	3 (2, 4)				334					3 (2, 3)							
Depressive symptoms, Range [0, 3]	18880	0.6 (0.4)				14314					0.5 (0.5)							
(Lack of) Religiosity, Range [1], [4]	18862	2.5 (1.0)				14114					3.0 (0.7)							
30-day Cigarette use, Range [0, 30]	18796	5 (10)				14275					9 (13)							
Illicit drug use	5593	30 (27, 32)				4580					34 (32, 36)							
12-mos Binge drinking, Range [0, 6]	18875	0.7 (1.3)				14262					1.3 (1.6)							
Childhood physical maltreatment, Range [0, 6]															14627			0.5 (1.3)
Childhood sexual abuse, Range [0, 6]															14651			0.1 (0.7)

PSE = Perceived Survival Expectations. Assessed via: “What are your chances of living to age 35?”.

a Unweighted sample size. Means, Percentages (95% Confidence Intervals) are weighted to be representative of adolescents in grades 7–12 in the US during the1994–1995 school year.

At Wave IV, 7.2% of respondents had engaged in suicide ideation in the past 12 months and 1.6% had attempted suicide (Table 2). Approximately 1 in 4 were current daily smokers, of whom about one-third (or 8% of the Add Health Wave IV population) smoked a pack or more a day. While 12% reported binge drinking at least weekly, fewer (2.3%) reported exceeding recommended daily limits for moderate drinking (i.e., up to one drink per day for women and up to two drinks per day for men) [29]. Self-reported past-year marijuana use was relatively common (23%) and higher than past-year use of other illicit substances (11%).

**Table 2:** Wave IV Outcomes, Add Health.

	n	% (95% CI)^a^
12-mos Suicide ideation	972	7.2 (6.5, 7.9)
12-mos Suicide attempt	203	1.6 (1.2, 2.0)
Exceeds daily limits for moderate drinking	333	2.3 (2.0, 2.6)
*Cigarette smoking*		
None	9468	61 (59, 63)
Less than daily	2063	14 (13, 15)
1–19 cigarettes/day	2164	16 (15, 18)
A pack of more a day	972	8 (7, 9)
*12-mos ≥ Illicit drug use (other than marijuana)*		
None	13339	89 (88, 90)
Monthly or less	784	6 (5, 7)
2–3 days/month	248	2 (2, 2)
Weekly or more	420	3 (3, 4)
*12-mos ≥ Binge drinking*		
None	7855	50 (48, 53)
Monthly or less	4009	28 (27, 30)
2–3 days/month	1243	9 (8, 10)
Weekly or more	1637	12 (11, 13)
*12-mos ≥ Marijuana use*		
None	11548	77 (75, 79)
Monthly or less	1368	9 (8, 10)
2–3 days/month	341	3 (2, 3)
Weekly or more	1522	11 (10, 12)

a Unweighted sample size. Percentages (95% Confidence Intervals) are weighted.

### PSE as a Predictor of Wave IV Suicidal Behavior

Low PSE at Waves I and III predicted greater risk of suicidal behavior at Wave IV. Reporting that one had a 50–50 chance or less of living to age 35 at Wave I or Wave III versus reporting that one was “almost certain” of living to age 35 was associated with a 29% and 44%, respectively, increase in risk of suicidal ideation at Wave IV (Table 3). Low PSE at Waves I or III was additionally related to approximately double the risk of suicide attempt at Wave IV. Even stronger associations were observed for individuals who reported persistently low PSE at Waves I and III. Compared to those who reported high PSE at Waves I and III, individuals who reported low PSE at both waves had twice the risk of suicidal ideation (RR: 1.93) and more than three times the risk of suicide attempt (RR: 3.39) at Wave IV (Table 3).

**Table 3:** Perceived Survival Expectations (PSE) as a predictor of Wave IV suicidal behavior, Add Health.

	Suicidal ideation		Suicide attempt	
	%	RR (95% CI)	%	RR (95% CI)
***Wave I PSE*** a	*N = 13581*		*N = 13585*	
≤ A 50–50 chance	9.8	1.29 (1.03, 1.62)	2.4	1.74 (1.00, 3.02)
A good chance	8.1	1.28 (1.05, 1.55)	1.7	1.48 (0.93, 2.35)
Almost certain	5.8	1.00	1.0	1.00
***Wave III PSE*** b	*N = 10675*		*N = 10676*	
≤ A 50–50 chance	13.1	1.44 (1.05, 1.98)	3.1	2.16 (0.96, 4.87)
A good chance	7.8	1.13 (0.89, 1.43)	1.5	1.47 (0.85, 2.55)
Almost certain	6.1	1.00	1.0	1.00
**Persistence of low PSE** c	*N = 11305*		*N = 11306*	
Low PSE at both Waves	14.7	1.93 (1.08, 3.46)	5.1	3.39 (1.02, 11.26)
High PSE at both Waves	6.2	1.00	1.1	1.00

PSE = Perceived Survival Expectations. Assessed via: “What are your chances of living to age 35?”.

Unweighted sample size; Percentages, Relative Risks (95% Confidence Intervals) are weighted.

a Log-binomial regression model controlled for age, sex, race/ethnicity, foreign-birth, parental education, family structure, childhood physical maltreatment, childhood sexual abuse and Wave I values for block group poverty, family history of suicide, history of suicide among friends, depressive symptoms, religiosity, parental attachment/support, cigarette smoking, binge drinking, illicit drug use and self-rated health.

b Log-binomial regression model controlled for the above-listed Wave III equivalent covariates.

c Log-binomial regression model controlled for the above-listed Wave I equivalent covariates.

Substance use has been shown to increase the risk of suicidal attempts [35]. Nonetheless, associations between PSE and Wave IV suicidal behavior persisted even after controlling for concurrent (Wave IV) substance use including marijuana use, other illicit substance use, binge drinking, heavy drinking, and smoking (results not shown). In multivariate analyses, Waves I/III depressive symptoms, illicit drug use, history of suicide among family or friends and experience of physical or sexual child maltreatment were associated with increased risk of suicide ideation at Wave IV. Waves I/III illicit drug use and history of suicide among family or friends predicted suicide attempt at Wave IV (Table S3).

### PSE as a Predictor of Wave IV Substance Use

Low PSE was associated with drinking, smoking, and illicit substance use at Wave IV, but only at relatively high levels of use. To conserve space, we only show a subset of relationships in Tables 4 and 5. The uncondensed tables are available in Tables S4, S5, S6, S7, S8. Associations between PSE and substance use were moderately attenuated after controlling for previous substance use. For instance, the adjusted odds ratios (AORs) for Wave I PSE ≤50% not controlling for previous substance use were 1.95, 1.79, and 1.68 for exceeding daily limits for moderate drinking, smoking at least a pack a day, and using illicit substances other than marijuana at least weekly, respectively. After controlling for previous substance use the respective AORs were 1.74, 1.62, and 1.46. Individuals with low PSE at both Waves I and III were about 2 times as likely to exceed daily limits for moderate drinking, 2.5 times as likely to smoke at least a pack a day, and 3.75 times as likely to use illicit substances other than marijuana at least weekly compared to those who reported high PSE at both waves (Table 4).

**Table 4:** Perceived Survival Expectations (PSE) as a predictor of Wave IV heavy drinking, smoking and illicit substance use, Add Health.

	Log binomial regression (Dichotomous outcome)		Multinomial logistic regression (Polytomous outcome, only one comparison shown)			
	Exceeds recommended daily limits for moderate drinking		Smoking at least a pack a day (≥20 cigarettes) vs. None		Illicit substance use (other than marijuana) ≥ Weekly vs. None	
	%	RR (95% CI)	%	AOR (95% CI)	%	AOR (95% CI)
***Wave I PSE*** a	*N = 13872*		*N = 13825*		*N = 13884*	
≤ A 50–50 chance	4.0	1.95 (1.27, 2.99)	12.6	1.79 (1.35, 2.37)	4.6	1.68 (1.20, 2.35)
A good chance	2.0	0.93 (0.65, 1.33)	8.0	1.07 (0.80, 1.42)	3.5	1.38 (1.04, 1.82)
Almost certain	2.1	1.00	7.2	1.00	2.4	1.00
***Wave III PSE*** b	*N = 11065*		*N = 11032*		*N = 11074*	
≤ A 50–50 chance	2.6	1.12 (0.61, 2.07)	14.3	1.65 (1.08, 2.52)	6.9	2.17 (1.34, 3.50)
A good chance	2.5	1.15 (0.78, 1.70)	7.8	0.97 (0.75, 1.26)	2.9	0.98 (0.60, 1.60)
Almost certain	2.0	1.00	7.4	1.00	2.5	1.00
**Persistence of low PSE** c	*N = 11522*		*N = 11487*		*N = 11530*	
Low PSE at both Waves	3.5	1.92 (0.80, 4.61)	15	2.53 (1.42, 4.51)	8.5	3.75 (1.85, 7.62)
High PSE at both Waves	1.9	1.00	7	1.00	2.4	1.00

PSE = Perceived Survival Expectations. Assessed via: “What are your chances of living to age 35?”.

Unweighted sample size, All other estimates are weighted.

a Model controlled for age, sex, race/ethnicity, foreign-birth, parental education, family structure, childhood physical abuse, childhood sexual abuse and Wave I values for block group poverty, depressive symptoms, religiosity, parental attachment/support and self-rated health.

b Model controlled for the above-listed Wave III covariates.

c Model controlled for the above-listed Wave I covariates.

**Table 5:** Perceived Survival Expectations (PSE) as a predictor of Wave IV binge drinking and marijuana use, Add Health.

	Multinomial logistic regression (Polytomous outcome, only one comparison shown)			
	Binge drinking (≤ Monthly vs. None)		Marijuana use (≤ Monthly vs. None)	
	%	AOR (95% CI)	%	AOR (95% CI)
***Wave I PSE*** a	*N = 13853*		*N = 13876*	
≤ A 50–50 chance	21	0.72 (0.60, 0.87)	7	0.81 (0.61, 1.07)
A good chance	30	1.07 (0.95, 1.20)	9	0.98 (0.81, 1.18)
Almost certain	31	1.00	10	1.00
***Wave III PSE*** b	*N = 11053*		*N = 11069*	
≤ A 50–50 chance	22	0.73 (0.55, 0.96)	10	1.17 (0.82, 1.67)
A good chance	27	0.91 (0.78, 1.07)	8	0.82 (0.63, 1.06)
Almost certain	31	1.00	10	1.00
**Persistence of low PSE** c	*N = 11507*		*N = 11524*	
Low PSE at both Waves	18	0.65 (0.40, 1.04)	8	1.03 (0.51, 2.07)
High PSE at both Waves	31	1.00	10	1.00

PSE = Perceived Survival Expectations. Assessed via: “What are your chances of living to age 35?”.

Unweighted sample size; Percentages, Adjusted Odds Ratio (95% Confidence Intervals) are weighted.

a Model controlled for age, sex, race/ethnicity, foreign-birth, parental education, family structure, childhood physical abuse, childhood sexual abuse and Wave I values for block group poverty, depressive symptoms, religiosity, parental attachment/support and self-rated health.

b Model controlled for the above-listed Wave III covariates.

c Model controlled for the above-listed Wave I covariates.

However, low Wave I (AOR: 0.72) and III PSE (AOR: 0.73) were related to lower binge drinking at a rate of monthly or less (Table 5). In multivariate models, younger age, male sex, previous substance use and lack of religiosity were related to higher substance use at Wave IV. Blacks were less likely to report exceeding daily limits for moderate drinking, binge drinking, and illicit drug use (other than marijuana) than white, non-Hispanics. The patterning of relationships between Waves I/III depressive symptoms and substance use at Wave IV was similar to that seen for low PSE. For instance, higher depressive symptoms suggested greater adjusted odds of smoking at least a pack a day and using illicit substances other than marijuana at least monthly.

**Figure 1:**
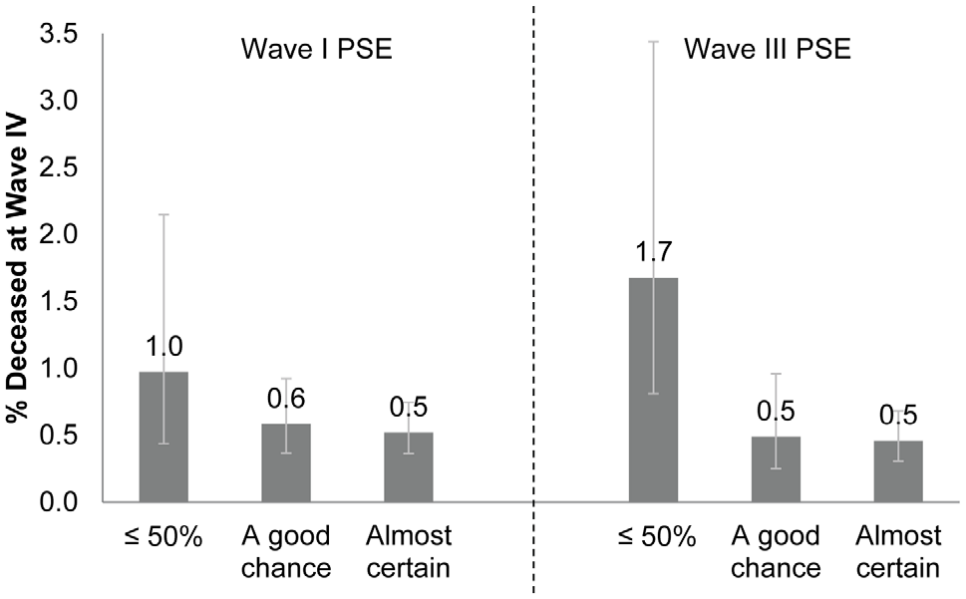
Percent deceased (n = 131) by Wave I and Wave III PSE.

We examined Wave I and Wave III PSE as predictors of actual mortality risk in young adulthood by assessing ties between PSE and death occurring between Waves III and IV (n = 131) (Figure 1). Individuals who reported Wave I PSE ≤50% experienced death rates that were double those experienced by individuals reporting “almost certain” (1% vs. 0.5%; crude OR: 1.88; 95% CI: 0.77, 4.56). Wave III PSE ≤50% was associated with a more than tripling of the odds of death at Wave IV (1.7% vs. 0.5%; crude OR: 3.70; 95% CI: 1.74, 7.90). While controlling for age, sex, and race/ethnicity attenuated the relationship between Wave III PSE and Wave IV death, suggestion of strong relationship remained after adjustment (AOR: 3.00; 95% CI: 1.39, 6.48).

## Discussion

To the authors’ knowledge, this is the first study to demonstrate that adolescent expectations of early death can have long-lasting associations with risk behaviors over a decade later, independent of depressive symptoms and other important confounding individual and contextual factors. PSE ≤50% was linked to increased suicidal ideation and suicide attempt in young adulthood, and these associations persist even after accounting for depressive symptoms, history of suicide among family and friends, and concurrent substance use in addition to demographic and family characteristics. Thus, low PSE may uniquely contribute to identifying at-risk youth beyond traditionally assessed risk factors.

Moreover, this study is also the first to explore ties between PSE and varying levels of both legal and illegal substance use. Detrimental associations between low PSE and increased substance use were confined to heavy/regular use rather than occasional or moderate substance use. Specifically, low PSE predicted smoking at least a pack a day; consuming more than the recommended daily limits for moderate drinking; and using illicit substances other than marijuana at least weekly at Wave IV. Furthermore, ties between PSE and adult substance use endure even after controlling for previous substance use–suggesting that PSE may continue to be related to future adult risk behaviors above-and-beyond its association with adolescent risk-taking.

Although Wave III PSE was closer in time to Wave IV outcomes than Wave I PSE, the pattern and magnitude of associations with Wave IV outcomes were similar for Wave I and III PSE. However, only low Wave I PSE predicted exceeding the daily limits for moderate drinking at Wave IV–highlighting the importance of examining adolescent developmental factors on adult outcomes [36]. Earlier life factors may be important to the prediction of adult alcohol use given high rates of experimentation and opportunity for the development of substance use disorders in adolescence. For instance, 40% of adult alcoholics experience symptoms of alcoholism between 15 and 19 [36].

Unexpectedly, in this study we found that low PSE at Wave I were related to lower frequency of binge drinking. The complexity of motivational factors underlying substance use may help explain the patterning of associations. Episodic binge drinking among young adults may occur with the expectation of positive affective consequences of drinking. Indeed, social drinking motives (e.g., drinking to have fun with friends) are the most commonly endorsed drinking motives [37]. These drinking motives may be less prevalent among individuals with low PSE, leading to lower rates of binge drinking. People with low PSE may be more socially isolated. In a recent study, Duke and colleagues found that low PSE at Waves I and II predicted lower odds of prosocial activities (i.e., hanging out with friends, shopping, creative activities) and civic engagement (i.e., volunteer/community service, political activities, voting) at Wave III [8]. Alternatively, drinking to cope with problems (e.g., avoid or escape from problems) often involves drinking alone and is most related to the manifestation of drinking problems and alcohol misuse [37], [38], [39]. Hence, PSE may predict only a subset of substance use behaviors (i.e., heavy use rather than recreational use). Nonetheless, substance use motives were not assessed in Add Health, and warrant further investigation.

### Study Findings in Context

Our results are consistent with nascent literature that links anticipation of an early death among youth to a broad array of detrimental outcomes including suicidal behavior, participation in illegal activities, fight-related injury, and unsafe sexual activity [2], [3], [4]. Nevertheless, these studies examined outcomes among adolescents or individuals in their early twenties. This is the first study to connect PSE with risk behaviors over a decade later–important because it suggests that low PSE may have long-lasting associations with harmful outcomes.

This study also breaks new ground in research examining PSE and varying types of substance use. An Add Health study found that adolescents who reported low PSE at Waves I and II were more likely to report past-year illicit substance use at Wave III compared to those with high PSE at both waves, although effect measures fell short of statistical significance [8]. Additionally, Borowsky et al. found that Wave I PSE was unrelated to past-year illicit substance use at Waves II and III [4]. Previous Add Health studies may have had difficulty detecting a relationship between low PSE and illicit substance use because marijuana, the most common illicit substance, was not differentiated from other substances. In addition, level of substance use had only been examined as any past-year use. We found that low PSE predicted heavy/regular use of legal and illegal substances rather than low levels of substance use. We also connected low PSE to Wave IV death– which contrasts with the results of an earlier study based on Add Health data that reported a null relationship between low Wave I PSE and Wave III death [4]. This earlier study may have been limited by lower power given that only 96 respondents were confirmed deceased by Wave III.

In addition to risk behaviors, low PSE has been related to lower education attainment, personal earnings, physical activity, engagement with the community, self-esteem and life satisfaction [8], [40]. The breadth of ties between low PSE and future outcomes suggest that its relationship is not specific to one set of outcomes. Rather low PSE is predictive of many different adverse outcomes. In addition, the origins of low PSE are diverse–reflecting lived experiences, exposures, and resources such as violence involvement, self-esteem, depressive symptoms, adult and peer connection as well as racial/minority status, foreign-birth, and low parental socioeconomic status [4], [32]. Thus potential modes of intervention on low PSE are diverse.

At Wave I, close to a quarter of youth with parents receiving public assistance reported low PSE compared to 13% of youth whose parents did not receive public assistance [4]. Although an individual’s racial status, foreign-birth or family socioeconomic status cannot be altered or easily changed, the higher prevalence of low PSE among these groups suggest that designing interventions which target the healthy youth development of at-risk groups may narrow health inequities. These interventions may take the form of mentoring programs that connect youth with professionals in their prospective career of interest; college-readiness programs; tutoring programs; after-school recreational programs; summer camp or a variety of other programs that help keep youths engaged and support them in the completion of their goals.

Two youth programs that have overwhelming success include the Stanford Medical Youth Science Program (SMYSP) and BUILD. SMYSP organizes an annual, intensive summer residential program for low-income and minority high school students. SMYSP participants attend lectures given by Stanford professors, participate in anatomy labs and hospital internships, and go on weekly outings with mentors. To date, 100% of SMYSP participants have graduated high school; 84% have graduated from 4-year colleges (five times the rate of other low-income youth in California); and 47% have attended medical school or graduate school [41]. Alternatively, BUILD is an educational non-profit combating low high school graduation rates across urban communities in the United States by “using entrepreneurship to excite and empower disengaged, low-income youth through high school and college success.” To date, 100% of their program graduates have completed high school and been accepted to college [42]. A meta-analysis of mentoring program suggests that ongoing training of mentors, structured activities, involvement of parents, and monitoring of program implementation increases achievement of program goals [43].

### Study Strengths and Limitations

Using nationally representative, longitudinal data, this study found that anticipation of an early death was predictive of select health risk behaviors 6–13 years later among young adults aged 24–32 years. Nonetheless, this study is subject to several limitations. We were unable to assess the influence of numeracy on the reporting of PSE [44]. Our ability to examine the relationship between PSE and suicidal behavior was constrained by the exclusion of people who completed suicide. Respondents self-reported their substance use, which may be subject to under-reporting. If under-reporting is related to actual substance use (e.g., if heavier users under-report), this may attenuate observed relationships between PSE and substance use. However, Add Health used Computer Assisted Self-Interviewing (CASI) on sensitive questions pertaining to suicide and substance use, which has been shown to increase reporting of drug use and violence compared to a more standard self-administered questionnaire [45]. Moreover, our study focused on the examination of prevalent substance use behaviors (which are themselves associated with high health costs) rather than the onset of substance use disorders at Wave IV. It is possible that loss to follow-up may have biased observed relationships although the distribution of demographic characteristics was similar at Wave I and Wave IV, and survey weights adjust for attrition across waves.

### Study Implications

Future areas of research include delineation of how low PSE relates to future outcomes. In particular, further understanding of motivational factors for substance use that correlate with low PSE is needed. Moreover, further research is needed on how to successfully intervene on low PSE and related psychological factors associated with worse health outcomes. Beliefs individuals have about their futures may influence their decision-making. People with fatalistic perceptions of the future may not see the point of trying; they may be more reckless with themselves and others. Anticipation of an early death is not a typical health survey question. However, its demonstrated prevalence among adolescents and its long-term ties to wide-ranging unfavorable outcomes suggest it may contribute to identifying at-risk youth beyond measures of depressive symptoms, individual and family sociodemographics, and even previous risk behaviors. PSE may be incorporated into health and counseling sessions with adolescents. Positive adult connections and increases in self-esteem have been linked with transitioning out of having expectations of an early death [46]. Interventions may decrease hopelessness cognitions and promote positive future orientations by contesting interpretations of events viewed through a pessimistic inferential style and aiding in the development of problem-solving and coping abilities [47]. Efforts to delay the onset of alcohol and drug use during adolescence; reduce the quantity consumed; and otherwise alter its pattern of use so as to attenuate its riskiness would lower the burden of substance use and related outcomes [48]. The emerging literature on PSE highlights the importance of earlier life factors on adult health and behaviors.

## Supporting Information

Table S1 Variable definitions.
(DOCX)

Table S2 Descriptive Statistics, Add Health.
(DOCX)

Table S3 Perceived Survival Expectations (PSE) as a predictor of Wave IV suicidal ideation and attempt, Add Health.
(DOCX)

Table S4 Perceived Survival Expectations (PSE) as a predictor of exceeding daily limits for moderate drinking at Wave IV, Add Health.
(DOCX)

Table S5 Perceived Survival Expectations (PSE) as a predictor of smoking at least a pack/day Wave IV, Add Health.
(DOCX)

Table S6 Perceived Survival Expectations (PSE) as a predictor of illicit drug use (other than marijuana) at Wave IV, Add Health.
(DOCX)

Table S7 Perceived Survival Expectations (PSE) as a predictor of binge drinking at Wave IV, Add Health.
(DOCX)

Table S8 Perceived Survival Expectations (PSE) as a predictor of marijuana use at Wave IV, Add Health.
(DOCX)
