# Supplementary material for: Adolescent Expectations of Early Death Predict Adult Risk Behaviors
Source: PLoS One. 2012 Aug 1;7(8):e41905. doi: 10.1371/journal.pone.0041905 (PMC3411584; doi:10.1371/journal.pone.0041905)
Supplement: Table S1 — Variable definitions. (DOCX) [file pone.0041905.s001.docx]

| Table S1. Variable definitions | | | |
| --- | --- | --- | --- |
| **Variable** | **Question** | **Responses/Coding** | **Waves utilized** |
| Perceived Survival Expectations (PSE) | What are your chances of living to age 35? | Almost no chance; Some chance, but probably not; A 50-50 chance; A good chance; Almost certain | I, II, III |
| **Covariates** |  |  |  |
| Poverty rate | Wave I: Census of Population and Housing 1990; Wave III: Census of Population and Housing 2000 | Block group level; % | I, III |
| Age |  | Years | I, III, IV |
| Sex |  | Male/female | I |
| Race/ethnicity | 1) What is your Hispanic or Latino background? 2) What is your race? | White, non-Hispanic; Black, non-Hispanic; Asian, non-Hispanic; Other, non-Hispanic; Multiracial | I |
| Foreign-birth | Were you born a U.S. citizen? | Yes/no | I |
| Parent's education | Parent interview: How far did you go in school? How far did your current (spouse/partner) go in school? Respondent interview: How far in school did [residential mother/father] go? | Highest education achieved by mother or father; If parent interview missing (14%), use respondent's report. Less than high school; High school/GED, Some college/AA degree,  ≥ College | I |
| Family structure |  | Two biological parents; Two parents; Single parent/other | I |
| (Lack of) Parental attachment/support | Wave I: 1) Closeness to mother/father, 2) think mother/father is warm and loving, 3) think mother/father care about respondent, 4) satisfaction with communication, and 5) overall satisfaction with relationship; Wave III: 1) enjoy doing things with mother/father, 2) closeness to mother/father, and 3) think mother/father is warm and loving | Cronbach’s alpha for Wave I: 0.84; Cronbach’s alpha for Wave III: 0.83 | I, III |
| Childhood physical maltreatment | Before your 18th birthday, how often did a parent or adult caregiver hit you with a fist, kick you, or throw you down on the floor, into a wall, or down stairs | 0 = None; 1= One time; 2 = 2 times; 3 = 3 to 5 times; 4 = 6 to 10 times; 5= >10 times | IV |
| Childhood sexual maltreatment | Before your 18th birthday, how often did a parent or other adult caregiver touch you in a sexual way, force you to touch him or her in a sexual way, or force you to have sexual relations? | 0 = None; 1= One time; 2 = 2 times; 3 = 3 to 5 times; 4 = 6 to 10 times; 5= >10 times | IV |
| Family history of suicide | 1) Have any of your family members tried to kill themselves during the past 12 months? | 1=Suicide attempt resulted in death; 2= Suicide attempt but no death (referent = No suicide attempt) | I, III |
|  | 2) Have any of them died as a result? |  |  |
| History of suicide among friends | 1) Have any of your friends tried to kill themselves during the past 12 months? 2) Have any of them died as a result? | 1=Suicide attempt resulted in death; 2= Suicide attempt but no death (referent = No suicide attempt) | I, III |
| Depressive symptoms | Wave I: 17 Center for Epidemiologic Studies Depression Scale items (CES-D); Wave III: 9 CES-D items | Cronbach’s alpha for Wave I: 0.86; Cronbach’s alpha for Wave III: 0.80 | I, III |
| (Lack of) Religiosity | 1) attendance of religious services, 2) attendance of activities offered by places of worship (i.e., choir, Bible classes), 3) importance of religion | Cronbach’s alpha for Wave I: 0.70; Cronbach’s alpha for Wave III: 0.75 | I, III |
| Self-rated health | In general, how is your health? | Fair/poor; Good; Very good; Excellent | I, III |
| 30-day Cigarette use | During the past 30 days, on how many days did you smoke cigarettes? | 0 to 30 days | I, III |
| Illicit drug use | Wave I: How old were you when you tried 1) marijuana 2) cocaine, 3) any other type of illegal drug? Wave III: In the past year, have you used 1) marijuana, 2) cocaine, 3) crystal meth, 4) any other type of illegal drugs? | Wave I: Any lifetime illicit drug use vs. None; Wave III: Any 12-month illicit drug use vs. None | I, III |
|  |  |  |  |
|  |  |  |  |
| 12-month Binge drinking | Over the past 12 months, on how many days did you drink five or more drinks in a row? | Range 0 (never) to 6 (every day or almost every day) | I, III |
| **Wave IV Outcomes** |  |  |  |
| Suicide ideation | During the past 12 months, have you ever seriously thought about committing suicide? | Yes/no | IV |
| Suicide attempt | During the past 12 months, how many times have you actually attempted suicide? | Any vs. None | IV |
| Cigarette smoking | 1) During the past 30 days, on how many days did you smoke cigarettes? 2) During the past 30 days, on the days you smoked, how many cigarettes did you smoke each day? | None; Less than daily smoking; Daily smoking (1 to 19 cigarettes a day; Smoking at least a pack a day (≥ 20 cigarettes) | IV |
|  |  |  |  |
|  |  |  |  |
| 12-month illicit substance use (other than marijuana) | During the past 12 months, on how many days did you use favorite drug? (excludes marijuana) | None; ≤ Monthly; 2-3 Days a month; ≥ Weekly | IV |
|  |  |  |  |
|  |  |  |  |
| Exceeds daily limits for moderate drinking^a^ | 1) During the past 12 months, on how many days did you drink alcohol? 2) How many drinks did you usually have each time? | Yes/No; Women: 2 or more drinks daily; Men: 3 or more drinks daily | IV |
| 12-month Binge drinking | During the past 12 months, on how many days did you 5/4 [men/women] or more drinks in a row? | None; ≤ Monthly; 2-3 Days a month; ≥ Weekly | IV |
|  |  |  |  |
|  |  |  |  |
| 12-month Marijuana use | During the past 12 months, on how many days did you use marijuana? | None; ≤ Monthly; 2-3 Days a month; ≥ Weekly | IV |
|  |  |  |  |
|  |  |  |  |
| ^a^ Department of Health and Human Services and the Department of Agriculture. (2005). Dietary Guidelines for Americans 2005 | | | |
