# Supplementary material for: Adolescent Expectations of Early Death Predict Adult Risk Behaviors
Source: PLoS One. 2012 Aug 1;7(8):e41905. doi: 10.1371/journal.pone.0041905 (PMC3411584; doi:10.1371/journal.pone.0041905)
Supplement: Table S2 — Descriptive Statistics, Add Health. (DOCX) [file pone.0041905.s002.docx]

| Table S2. Descriptive Statistics, Add Health | | | | | | |  |  |  |  |
| --- | --- | --- | --- | --- | --- | --- | --- | --- | --- | --- |
|  | | **Wave I (1994-95)** | | **Wave II (1996)** | | **Wave III (2001-02)** | | | **Wave IV (2008)** | |
|  | | **n** | **% (95% CI) or Mean (SD)^a^** | **n** | **% (95% CI) or Mean (SD)^a^** | **n** | **% (95% CI) or Mean (SD)^a^** | | **n** | **% (95% CI) or Mean (SD)^a^** |
| **Perceived survival expectations** | |  |  |  |  |  |  | |  |  |
| Almost certain | | 10250 | 57 (55, 59) | 7016 | 54 (52, 56) | 10309 | 74 (72, 75) | |  |  |
| A good chance | | 5775 | 29 (28, 30) | 4336 | 31 (29, 32) | 2817 | 19 (18, 20) | |  |  |
| A 50-50 chance | | 2065 | 11 (10, 12) | 1670 | 12 (11, 13) | 1020 | 6.8 (6.1, 7.6) | |  |  |
| Some chance but probably not | | 439 | 2 (2, 3) | 325 | 2 (2, 3) | 63 | 0.4 (0.3, 0.5) | |  |  |
| Almost no chance | | 257 | 1 (1, 2) | 169 | 1 (1, 2) | 30 | 0.2 (0.1, 0.3) | |  |  |
| **Covariates** | |  |  |  |  |  |  | |  |  |
| Age (years) | | 18919 | 16 (2) |  |  |  |  | | 14800 | 28 (2) |
| Male | | 9288 | 51 (50, 52) |  |  |  |  | | 6930 | 51 (49, 52) |
| *Race* | |  |  |  |  |  |  | |  |  |
| White, non-Hispanic | | 9608 | 65 (59, 70) |  |  |  |  | | 7849 | 66 (60, 71) |
| Black, non-Hispanic | | 3790 | 15 (11, 19) |  |  |  |  | | 2975 | 15 (12, 20) |
| Hispanic | | 2993 | 11 (8, 15) |  |  |  |  | | 2168 | 11 (8, 15) |
| Asian, non-Hispanic | | 1247 | 3 (2, 5) |  |  |  |  | | 836 | 3 (2, 5) |
| Other, non-Hispanic | | 270 | 1 (1, 2) |  |  |  |  | | 182 | 1 (1,2) |
| Multiracial | | 936 | 4 (3, 5) |  |  |  |  | | 729 | 4 (3, 5) |
| Foreign-born | | 1746 | 6 (5, 9) |  |  |  |  | | 1129 | 5 (4, 7) |
| *Parent's education* | |  |  |  |  |  |  | |  |  |
| Less than high school | | 2527 | 13 (10, 15) |  |  |  |  | |  |  |
| High school/GED | | 4704 | 28 (25, 30) |  |  |  |  | |  |  |
| Some college/AA degree | | 5333 | 30 (28, 31) |  |  |  |  | |  |  |
| College or greater | | 6011 | 30 (27, 34) |  |  |  |  | |  |  |
| *Family structure* | |  |  |  |  |  |  | |  |  |
| Two biological parents | | 9686 | 53 (51, 56) |  |  |  |  | |  |  |
| Two parents | | 3444 | 17 (16, 18) |  |  |  |  | |  |  |
| Single parent/other | | 5794 | 29 (27, 32) |  |  |  |  | |  |  |
| Block group poverty rate | | 18627 | 12 (13) |  |  | 14051 | 15 (13) | |  |  |
| Parental support, Range [1,5] | | 18526 | 1.6 (0.6) |  |  | 13661 | 1.5 (0.7) | |  |  |
| *12-mos family history of suicide* | |  |  |  |  |  |  | |  |  |
| No suicide attempt | | 17862 | 95 (95, 96) |  |  | 13517 | 97 (97, 98) | |  |  |
| Suicide attempt | | 658 | 4 (3, 4) |  |  | 290 | 2 (2, 2) | |  |  |
| Suicide attempt resulted in death | | 170 | 1 (1, 1) |  |  | 100 | 1 (1, 1) | |  |  |
| *12-mos history of suicide among friends* | |  |  |  |  |  |  | |  |  |
| No suicide attempt | | 15429 | 82 (81, 83) |  |  | 12963 | 93 (92, 93) | |  |  |
| Suicide attempt | | 2690 | 15 (14, 16) |  |  | 601 | 4 (4, 5) | |  |  |
| Suicide attempt resulted in death | | 542 | 3 (2, 4) |  |  | 334 | 3 (2, 3) | |  |  |
| Depressive symptoms, Range [0, 3] | | 18880 | 0.6 (0.4) |  |  | 14314 | 0.5 (0.5) | |  |  |
| (Lack of) Religiosity, Range [1, 4] | | 18862 | 2.5 (1.0) |  |  | 14114 | 3.0 (0.7) | |  |  |
| *Self-rated health* | |  |  |  |  |  |  | |  |  |
| Fair/poor | | 1339 | 7 (6, 8) |  |  | 650 | 5 (4, 5) | |  |  |
| Good | | 4832 | 26 (25, 27) |  |  | 3149 | 22 (21, 23) | |  |  |
| Very good | | 7431 | 39 (38, 40) |  |  | 5805 | 41 (40, 42) | |  |  |
| Excellent | | 5300 | 28 (27, 29) |  |  | 4716 | 32 (31, 34) | |  |  |
| 30-day Cigarette use, Range [0, 30] | | 18796 | 5 (10) |  |  | 14275 | 9 (13) | |  |  |
| Illicit drug use | | 5593 | 30 (27, 32) |  |  | 4580 | 34 (32, 36) | |  |  |
| 12-mos Binge drinking, Range [0, 6] | | 18875 | 0.7 (1.3) |  |  | 14262 | 1.3 (1.6) | |  |  |
| Childhood physical maltreatment, Range [0, 6] | |  |  |  |  |  |  | | 14627 | 0.5 (1.3) |
| Childhood sexual abuse, Range [0, 6] | |  |  |  |  |  |  | | 14651 | 0.1 (0.7) |
| PSE= Perceived Survival Expectations. Assessed via: "What are your chances of living to age 35?" | | | | | | | | | | |
| ^a^ Unweighted sample size. Means, Percentages (95% Confidence Intervals) are weighted to be representative of adolescents in grades 7–12 in the US during the1994–1995 school year. | | | | | | | | | | |
