# Supplementary material for: Adolescent Expectations of Early Death Predict Adult Risk Behaviors
Source: PLoS One. 2012 Aug 1;7(8):e41905. doi: 10.1371/journal.pone.0041905 (PMC3411584; doi:10.1371/journal.pone.0041905)
Supplement: Table S3 — Perceived Survival Expectations (PSE) as a predictor of Wave IV suicidal ideation and attempt, Add Health. (DOCX) [file pone.0041905.s003.docx]

| Table S3. Perceived Survival Expectations (PSE) as a predictor of Wave IV suicidal ideation and attempt, Add Health | | |
| --- | --- | --- |
|  | **Wave I** | **Wave III** |
|  | **AOR (95% CI)^b^** | **AOR (95% CI)^b^** |
|  | **Suicide ideation** | |
| Wave I/III PSE ≤ 50% | 1.29 (1.03, 1.62) | 1.44 (1.05, 1.98) |
| Wave I/III PSE "A good chance" | 1.28 (1.05, 1.55) | 1.13 (0.89, 1.43) |
| Age (years) | 0.96 (0.91, 1.02) | 1.00 (0.94, 1.06) |
| Male | 1.15 (0.93, 1.41) | 1.13 (0.92, 1.39) |
| Foreign-born (vs. US-born) | 1.02 (0.67, 1.56) | 1.08 (0.66, 1.79) |
| Black, non-Hispanic (vs. white, non-Hispanic) | 0.84 (0.64, 1.10) | 0.95 (0.72, 1.27) |
| Hispanic (vs. white, non-Hispanic) | 0.71 (0.43, 1.18) | 0.60 (0.42, 0.86) |
| Asian, non-Hispanic (vs. white, non-Hispanic) | 0.84 (0.52, 1.36) | 0.85 (0.49, 1.46) |
| Multiracial, non-Hispanic (vs. white, non-Hispanic) | 0.94 (0.60, 1.48) | 0.91 (0.57, 1.47) |
| Other, non-Hispanic (vs. white, non-Hispanic) | 0.67 (0.34, 1.35) | 0.85 (0.44, 1.64) |
| Parental education < high school (vs. ≥ college) | 1.13 (0.84, 1.53) | 1.45 (1.06, 1.99) |
| Parental education high school or GED (vs. ≥ college) | 0.94 (0.72, 1.21) | 0.99 (0.76, 1.30) |
| Parental education some college (vs. ≥ college) | 0.94 (0.75, 1.18) | 1.03 (0.80, 1.33) |
| Wave I/III Block group poverty rate, % | 1.00 (0.99, 1.01) | 1.00 (0.99, 1.01) |
| Family structure: Two parents (vs. two biological parents) | 1.14 (0.76, 1.71) | 0.99 (0.79, 1.24) |
| Family structure: Single parent/other (vs. two biological parents) | 2.14 (1.18, 3.86) | 1.13 (0.91, 1.39) |
| Wave I/III Parental attachment/support | 0.98 (0.89, 1.09) | 1.00 (0.96, 1.04) |
| Childhood physical maltreatment | 1.13 (1.07, 1.20) | 1.17 (1.11, 1.25) |
| Childhood sexual abuse | 1.22 (1.13, 1.32) | 1.15 (1.05, 1.26) |
| 12-mos family history of suicide: Family member attempted suicide (vs. no history) | 1.14 (0.76, 1.71) | 0.79 (0.45, 1.38) |
| 12-mos family history of suicide: Suicide attempt resulted in death (vs. no history) | 2.14 (1.18, 3.86) | 2.00 (0.98, 4.09) |
| 12-mos history of suicide among friends: Friend attempted suicide | 1.50 (1.23, 1.83) | 1.42 (0.96, 2.10) |
| 12-mos history of suicide among friends: Suicide attempt resulted in death | 1.53 (1.06, 2.21) | 1.59 (1.00, 2.54) |
| (Lack of) Religiosity | 1.10 (1.01, 1.19) | 1.15 (0.97, 1.36) |
| Wave I/III Binge drinking | 0.90 (0.83, 0.99) | 0.91 (0.86, 0.97) |
| Wave I/III Cigarette smoking | 0.99 (0.98, 1.00) | 1.00 (1.00, 1.01) |
| Wave I/III Illicit drug use | 1.53 (1.21, 1.92) | 1.35 (1.08, 1.69) |
| Wave I/III Fair/poor self-rated health (vs. excellent) | 1.35 (0.98, 1.85) | 1.32 (0.87, 2.00) |
| Wave I/III Good self-rated health (vs. excellent) | 1.23 (0.93, 1.64) | 1.16 (0.87, 1.55) |
| Wave I/III Very good self-rated health (vs. excellent) | 1.16 (0.87, 1.55) | 1.10 (0.84, 1.43) |
| Wave I/III Depressive symptoms | 1.81 (1.49, 2.21) | 1.78 (1.49, 2.13) |
|  | **Suicide attempt** | |
| Wave I/III PSE ≤ 50% | 1.74 (1.00, 3.02) | 2.16 (0.96, 4.87) |
| Wave I/III PSE "A good chance" | 1.48 (0.93, 2.35) | 1.47 (0.85, 2.55) |
| Age (years) | 1.01 (0.86, 1.17) | 1.05 (0.91, 1.22) |
| Male | 0.76 (0.44, 1.31) | 0.57 (0.36, 0.92) |
| Foreign-born (vs. US-born) | 0.54 (0.13, 2.26) | 0.95 (0.23, 3.97) |
| Black, non-Hispanic (vs. white, non-Hispanic) | 1.07 (0.53, 2.13) | 1.67 (0.90, 3.09) |
| Hispanic (vs. white, non-Hispanic) | 0.94 (0.23, 3.94) | 0.61 (0.25, 1.47) |
| Asian, non-Hispanic (vs. white, non-Hispanic) | 0.19 (0.04, 0.90) | 0.24 (0.04, 1.32) |
| Multiracial, non-Hispanic (vs. white, non-Hispanic) | 0.54 (0.21, 1.37) | 0.71 (0.27, 1.88) |
| Other, non-Hispanic (vs. white, non-Hispanic) | 0.30 (0.08, 1.16) | 1.01 (0.30, 3.40) |
| Parental education < high school (vs. ≥ college) | 1.35 (0.58, 3.14) | 2.36 (1.06, 5.24) |
| Parental education high school or GED (vs. ≥ college) | 0.74 (0.40, 1.38) | 0.92 (0.47, 1.78) |
| Parental education some college (vs. ≥ college) | 0.79 (0.39, 1.58) | 1.09 (0.60, 1.99) |
| Wave I/III Block group poverty rate, % | 1.01 (0.99, 1.03) | 1.00 (0.99, 1.02) |
| Family structure: Two parents (vs. two biological parents) | 1.15 (0.64, 2.07) | 1.48 (0.81, 2.68) |
| Family structure: Single parent/other (vs. two biological parents) | 0.78 (0.45, 1.32) | 0.87 (0.48, 1.58) |
| Wave I/III Parental attachment/support | 0.94 (0.72, 1.23) | 1.06 (0.98, 1.16) |
| Childhood physical maltreatment | 1.10 (0.97, 1.24) | 1.16 (1.00, 1.35) |
| Childhood sexual abuse | 1.17 (1.00, 1.38) | 1.12 (0.93, 1.36) |
| 12-mos family history of suicide: Family member attempted suicide (vs. no history) | 2.62 (1.45, 4.74) | 1.08 (0.35, 3.31) |
| 12-mos family history of suicide: Suicide attempt resulted in death (vs. no history) | 2.12 (0.56, 7.94) | 2.78 (0.71, 10.80) |
| 12-mos history of suicide among friends: Friend attempted suicide | 1.34 (0.86, 2.09) | 1.93 (0.89, 4.17) |
| 12-mos history of suicide among friends: Suicide attempt resulted in death | 2.90 (1.46, 5.78) | 2.57 (1.03, 6.40) |
| (Lack of) Religiosity | 0.91 (0.76, 1.10) | 1.62 (1.03, 2.54) |
| Wave I/III Binge drinking | 1.04 (0.86, 1.27) | 0.87 (0.71, 1.06) |
| Wave I/III Cigarette smoking | 0.98 (0.95, 1.00) | 1.02 (0.99, 1.04) |
| Wave I/III Illicit drug use | 1.63 (1.04, 2.56) | 1.83 (1.14, 2.95) |
| Wave I/III Fair/poor self-rated health (vs. excellent) | 1.22 (0.61, 2.44) | 1.47 (0.63, 3.43) |
| Wave I/III Good self-rated health (vs. excellent) | 1.05 (0.54, 2.02) | 0.69 (0.36, 1.31) |
| Wave I/III Very good self-rated health (vs. excellent) | 0.71 (0.40, 1.27) | 0.84 (0.43, 1.62) |
| Wave I/III Depressive symptoms | 1.37 (0.74, 2.55) | 1.10 (0.67, 1.81) |
