# Supplementary material for: Adolescent Expectations of Early Death Predict Adult Risk Behaviors
Source: PLoS One. 2012 Aug 1;7(8):e41905. doi: 10.1371/journal.pone.0041905 (PMC3411584; doi:10.1371/journal.pone.0041905)
Supplement: Table S4 — Perceived Survival Expectations (PSE) as a predictor of exceeding daily limits for moderate drinking at Wave IV, Add Health. (DOCX) [file pone.0041905.s004.docx]

| Table S4. Perceived Survival Expectations (PSE) as a predictor of exceeding daily limits for moderate drinking at Wave IV, Add Health | | |
| --- | --- | --- |
|  | **Wave I** | **Wave III** |
|  | **AOR (95% CI)^b^** | **AOR (95% CI)^b^** |
| Wave I/III PSE ≤ 50% | 1.95 (1.27, 2.99) | 1.12 (0.61, 2.07) |
| Wave I/III PSE "A good chance" | 0.93 (0.65, 1.33) | 1.15 (0.78, 1.70) |
| Age (years) | 1.01 (0.94, 1.09) | 0.97 (0.89, 1.07) |
| Male | 2.79 (1.95, 4.00) | 2.53 (1.69, 3.78) |
| Foreign-born (vs. US-born) | 0.79 (0.28, 2.22) | 0.87 (0.24, 3.16) |
| Black, non-Hispanic (vs. white, non-Hispanic) | 0.58 (0.36, 0.94) | 0.68 (0.44, 1.04) |
| Hispanic (vs. white, non-Hispanic) | 0.63 (0.38, 1.03) | 0.79 (0.46, 1.37) |
| Asian, non-Hispanic (vs. white, non-Hispanic) | 0.92 (0.24, 3.55) | 0.92 (0.22, 3.78) |
| Multiracial, non-Hispanic (vs. white, non-Hispanic) | 1.14 (0.60, 2.17) | 1.23 (0.58, 2.59) |
| Other, non-Hispanic (vs. white, non-Hispanic) | 0.85 (0.29, 2.51) | 0.77 (0.17, 3.41) |
| Parental education < high school (vs. ≥ college) | 0.50 (0.32, 0.78) | 0.56 (0.30, 1.04) |
| Parental education high school or GED (vs. ≥ college) | 0.54 (0.35, 0.82) | 0.66 (0.42, 1.04) |
| Parental education some college (vs. ≥ college) | 0.84 (0.61, 1.17) | 0.91 (0.60, 1.37) |
| Wave I/III Block group poverty rate, % | 1.00 (0.99, 1.01) | 1.00 (0.99, 1.01) |
| Family structure: Two parents (vs. two biological parents) | 0.82 (0.54, 1.23) | 0.93 (0.59, 1.46) |
| Family structure: Single parent/other (vs. two biological parents) | 1.24 (0.88, 1.76) | 1.24 (0.82, 1.87) |
| Wave I/III Parental attachment/support | 0.94 (0.73, 1.20) | 0.93 (0.85, 1.01) |
| Childhood physical maltreatment | 1.03 (0.93, 1.13) | 0.93 (0.83, 1.05) |
| Childhood sexual abuse | 1.08 (0.89, 1.30) | 0.90 (0.67, 1.20) |
| (Lack of) Religiosity | 1.11 (0.95, 1.30) | 1.81 (1.34, 2.45) |
| Wave I/III Fair/poor self-rated health (vs. excellent) | 1.68 (0.92, 3.06) | 1.55 (0.70, 3.42) |
| Wave I/III Good self-rated health (vs. excellent) | 1.33 (0.87, 2.05) | 1.37 (0.86, 2.18) |
| Wave I/III Very good self-rated health (vs. excellent) | 1.14 (0.80, 1.64) | 0.85 (0.55, 1.32) |
| Wave I/III Depressive symptoms | 1.23 (0.84, 1.81) | 1.39 (0.97, 1.98) |
