# Supplementary material for: Adolescent Expectations of Early Death Predict Adult Risk Behaviors
Source: PLoS One. 2012 Aug 1;7(8):e41905. doi: 10.1371/journal.pone.0041905 (PMC3411584; doi:10.1371/journal.pone.0041905)
Supplement: Table S5 — Perceived Survival Expectations (PSE) as a predictor of smoking at least a pack/day Wave IV, Add Health. (DOCX) [file pone.0041905.s005.docx]

| Table S5. Perceived Survival Expectations (PSE) as a predictor of smoking at least a pack/day Wave IV, Add Health | | |
| --- | --- | --- |
|  | **Wave I** | **Wave III** |
|  | **AOR (95% CI)^b^** | **AOR (95% CI)^b^** |
|  | **Less than daily smoking** | |
| Wave I/III PSE ≤ 50% | 1.09 (0.88, 1.35) | 1.15 (0.87, 1.52) |
| Wave I/III PSE "A good chance" | 0.98 (0.84, 1.15) | 1.07 (0.91, 1.26) |
| Age (years) | 0.91 (0.88, 0.95) | 0.92 (0.88, 0.96) |
| Male | 1.62 (1.38, 1.90) | 1.52 (1.28, 1.81) |
| Foreign-born (vs. US-born) | 0.60 (0.42, 0.85) | 0.72 (0.49, 1.07) |
| Black, non-Hispanic (vs. white, non-Hispanic) | 0.89 (0.71, 1.12) | 1.06 (0.83, 1.34) |
| Hispanic (vs. white, non-Hispanic) | 1.16 (0.95, 1.43) | 1.14 (0.88, 1.48) |
| Asian, non-Hispanic (vs. white, non-Hispanic) | 0.93 (0.62, 1.38) | 0.94 (0.60, 1.48) |
| Multiracial, non-Hispanic (vs. white, non-Hispanic) | 0.95 (0.67, 1.34) | 1.05 (0.72, 1.53) |
| Other, non-Hispanic (vs. white, non-Hispanic) | 1.32 (0.76, 2.28) | 1.39 (0.77, 2.50) |
| Parental education < high school (vs. ≥ college) | 0.95 (0.73, 1.23) | 1.01 (0.76, 1.35) |
| Parental education high school or GED (vs. ≥ college) | 0.98 (0.79, 1.22) | 1.00 (0.80, 1.26) |
| Parental education some college (vs. ≥ college) | 0.88 (0.70, 1.12) | 0.94 (0.75, 1.18) |
| Wave I/III Block group poverty rate, % | 1.00 (1.00, 1.01) | 1.00 (1.00, 1.01) |
| Family structure: Two parents (vs. two biological parents) | 1.18 (0.98, 1.41) | 1.21 (0.99, 1.49) |
| Family structure: Single parent/other (vs. two biological parents) | 1.20 (1.03, 1.39) | 1.11 (0.93, 1.32) |
| Wave I/III Parental attachment/support | 1.01 (0.89, 1.14) | 0.96 (0.92, 1.00) |
| Childhood physical maltreatment | 1.05 (0.99, 1.11) | 1.09 (1.02, 1.16) |
| Childhood sexual abuse | 1.06 (0.96, 1.18) | 1.06 (0.95, 1.20) |
| (Lack of) Religiosity | 1.08 (1.01, 1.17) | 1.44 (1.29, 1.60) |
| Wave I/III Fair/poor self-rated health (vs. excellent) | 1.04 (0.77, 1.39) | 1.07 (0.73, 1.57) |
| Wave I/III Good self-rated health (vs. excellent) | 1.06 (0.87, 1.28) | 1.28 (1.01, 1.62) |
| Wave I/III Very good self-rated health (vs. excellent) | 1.17 (1.01, 1.36) | 1.14 (0.96, 1.34) |
| Wave I/III Depressive symptoms | 1.39 (1.19, 1.62) | 1.32 (1.08, 1.60) |
|  | **Daily smoking (≤19 cigarettes/day)** | |
| Wave I/III PSE ≤ 50% | 1.18 (0.93, 1.50) | 1.02 (0.77, 1.34) |
| Wave I/III PSE "A good chance" | 0.98 (0.83, 1.16) | 1.06 (0.87, 1.31) |
| Age (years) | 0.91 (0.86, 0.95) | 0.93 (0.88, 0.98) |
| Male | 1.21 (1.04, 1.42) | 1.13 (0.95, 1.35) |
| Foreign-born (vs. US-born) | 0.39 (0.24, 0.64) | 0.49 (0.29, 0.85) |
| Black, non-Hispanic (vs. white, non-Hispanic) | 0.49 (0.38, 0.64) | 0.64 (0.49, 0.83) |
| Hispanic (vs. white, non-Hispanic) | 0.45 (0.35, 0.58) | 0.48 (0.33, 0.69) |
| Asian, non-Hispanic (vs. white, non-Hispanic) | 0.91 (0.54, 1.51) | 0.81 (0.51, 1.30) |
| Multiracial, non-Hispanic (vs. white, non-Hispanic) | 0.99 (0.76, 1.29) | 1.11 (0.79, 1.56) |
| Other, non-Hispanic (vs. white, non-Hispanic) | 1.53 (0.65, 3.61) | 1.44 (0.60, 3.47) |
| Parental education < high school (vs. ≥ college) | 1.37 (1.06, 1.76) | 1.42 (1.06, 1.91) |
| Parental education high school or GED (vs. ≥ college) | 1.35 (1.09, 1.67) | 1.43 (1.14, 1.79) |
| Parental education some college (vs. ≥ college) | 1.44 (1.20, 1.73) | 1.36 (1.11, 1.66) |
| Wave I/III Block group poverty rate, % | 1.01 (1.00, 1.02) | 1.00 (0.99, 1.00) |
| Family structure: Two parents (vs. two biological parents) | 1.68 (1.40, 2.01) | 1.69 (1.39, 2.06) |
| Family structure: Single parent/other (vs. two biological parents) | 1.48 (1.22, 1.80) | 1.45 (1.16, 1.81) |
| Wave I/III Parental attachment/support | 1.02 (0.92, 1.12) | 0.98 (0.95, 1.02) |
| Childhood physical maltreatment | 1.07 (1.01, 1.13) | 1.03 (0.96, 1.11) |
| Childhood sexual abuse | 1.04 (0.93, 1.15) | 1.00 (0.88, 1.13) |
| (Lack of) Religiosity | 1.17 (1.08, 1.26) | 1.56 (1.39, 1.75) |
| Wave I/III Fair/poor self-rated health (vs. excellent) | 1.98 (1.47, 2.65) | 1.97 (1.29, 3.00) |
| Wave I/III Good self-rated health (vs. excellent) | 1.71 (1.44, 2.04) | 2.07 (1.65, 2.59) |
| Wave I/III Very good self-rated health (vs. excellent) | 1.33 (1.12, 1.58) | 1.51 (1.23, 1.84) |
| Wave I/III Depressive symptoms | 1.25 (1.01, 1.53) | 1.28 (1.07, 1.53) |
|  | **Smoking ≥ a pack/day (20 cigarettes)** | |
| Wave I/III PSE ≤ 50% | 1.79 (1.35, 2.37) | 1.65 (1.08, 2.52) |
| Wave I/III PSE "A good chance" | 1.07 (0.80, 1.42) | 0.97 (0.75, 1.26) |
| Age (years) | 0.90 (0.85, 0.96) | 0.94 (0.88, 1.01) |
| Male | 2.81 (2.21, 3.56) | 2.88 (2.22, 3.74) |
| Foreign-born (vs. US-born) | 0.51 (0.25, 1.06) | 0.34 (0.10, 1.09) |
| Black, non-Hispanic (vs. white, non-Hispanic) | 0.15 (0.09, 0.24) | 0.23 (0.13, 0.41) |
| Hispanic (vs. white, non-Hispanic) | 0.19 (0.11, 0.35) | 0.26 (0.13, 0.52) |
| Asian, non-Hispanic (vs. white, non-Hispanic) | 0.12 (0.05, 0.29) | 0.07 (0.02, 0.23) |
| Multiracial, non-Hispanic (vs. white, non-Hispanic) | 0.47 (0.25, 0.89) | 0.54 (0.26, 1.13) |
| Other, non-Hispanic (vs. white, non-Hispanic) | 0.53 (0.11, 2.48) | 0.36 (0.08, 1.51) |
| Parental education < high school (vs. ≥ college) | 2.36 (1.54, 3.61) | 3.25 (2.03, 5.20) |
| Parental education high school or GED (vs. ≥ college) | 2.32 (1.84, 2.91) | 2.48 (1.89, 3.25) |
| Parental education some college (vs. ≥ college) | 1.86 (1.43, 2.40) | 1.87 (1.38, 2.54) |
| Wave I/III Block group poverty rate, % | 1.01 (1.00, 1.02) | 1.00 (0.99, 1.01) |
| Family structure: Two parents (vs. two biological parents) | 1.35 (1.03, 1.75) | 1.46 (1.07, 1.98) |
| Family structure: Single parent/other (vs. two biological parents) | 1.15 (0.90, 1.47) | 1.26 (0.95, 1.67) |
| Wave I/III Parental attachment/support | 1.01 (0.87, 1.18) | 0.98 (0.93, 1.04) |
| Childhood physical maltreatment | 1.14 (1.05, 1.23) | 1.11 (1.02, 1.20) |
| Childhood sexual abuse | 1.05 (0.89, 1.23) | 1.02 (0.82, 1.28) |
| (Lack of) Religiosity | 1.22 (1.11, 1.34) | 1.65 (1.38, 1.98) |
| Wave I/III Fair/poor self-rated health (vs. excellent) | 3.16 (2.27, 4.40) | 4.25 (2.76, 6.54) |
| Wave I/III Good self-rated health (vs. excellent) | 1.93 (1.47, 2.54) | 1.98 (1.43, 2.72) |
| Wave I/III Very good self-rated health (vs. excellent) | 1.29 (1.03, 1.61) | 1.38 (1.05, 1.80) |
| Wave I/III Depressive symptoms | 1.61 (1.26, 2.04) | 1.47 (1.11, 1.96) |
