# Supplementary material for: Adolescent Expectations of Early Death Predict Adult Risk Behaviors
Source: PLoS One. 2012 Aug 1;7(8):e41905. doi: 10.1371/journal.pone.0041905 (PMC3411584; doi:10.1371/journal.pone.0041905)
Supplement: Table S6 — Perceived Survival Expectations (PSE) as a predictor of illicit drug use (other than marijuana) at Wave IV, Add Health. (DOCX) [file pone.0041905.s006.docx]

| Table S6. Perceived Survival Expectations (PSE) as a predictor of illicit drug use (other than marijuana) at Wave IV, Add Health | | |
| --- | --- | --- |
|  | **Wave I** | **Wave III** |
|  | **AOR (95% CI)^b^** | **AOR (95% CI)^b^** |
|  | **≤ Monthly** | |
| Wave I/III PSE ≤ 50% | 0.95 (0.69, 1.32) | 0.98 (0.63, 1.53) |
| Wave I/III PSE "A good chance" | 1.06 (0.83, 1.35) | 0.97 (0.72, 1.31) |
| Age (years) | 0.89 (0.83, 0.95) | 0.89 (0.83, 0.95) |
| Male | 1.63 (1.32, 2.02) | 1.59 (1.27, 1.99) |
| Foreign-born (vs. US-born) | 0.49 (0.27, 0.90) | 0.44 (0.20, 0.96) |
| Black, non-Hispanic (vs. white, non-Hispanic) | 0.43 (0.30, 0.62) | 0.40 (0.28, 0.58) |
| Hispanic (vs. white, non-Hispanic) | 0.95 (0.66, 1.36) | 1.05 (0.70, 1.56) |
| Asian, non-Hispanic (vs. white, non-Hispanic) | 0.67 (0.33, 1.33) | 0.68 (0.31, 1.49) |
| Multiracial, non-Hispanic (vs. white, non-Hispanic) | 0.82 (0.50, 1.36) | 0.74 (0.44, 1.25) |
| Other, non-Hispanic (vs. white, non-Hispanic) | 1.19 (0.48, 2.92) | 1.49 (0.68, 3.25) |
| Parental education < high school (vs. ≥ college) | 0.78 (0.53, 1.15) | 0.61 (0.39, 0.96) |
| Parental education high school or GED (vs. ≥ college) | 0.84 (0.64, 1.09) | 0.72 (0.53, 0.97) |
| Parental education some college (vs. ≥ college) | 0.71 (0.55, 0.91) | 0.72 (0.55, 0.95) |
| Wave I/III Block group poverty rate, % | 0.99 (0.98, 1.00) | 1.00 (0.99, 1.01) |
| Family structure: Two parents (vs. two biological parents) | 1.23 (0.93, 1.64) | 1.14 (0.85, 1.52) |
| Family structure: Single parent/other (vs. two biological parents) | 1.39 (1.04, 1.86) | 1.38 (1.01, 1.87) |
| Wave I/III Parental attachment/support | 1.15 (0.97, 1.37) | 1.02 (0.96, 1.08) |
| Childhood physical maltreatment | 1.06 (0.98, 1.14) | 1.07 (0.98, 1.17) |
| Childhood sexual abuse | 1.10 (0.98, 1.24) | 1.13 (1.00, 1.28) |
| (Lack of) Religiosity | 1.24 (1.12, 1.37) | 1.62 (1.35, 1.96) |
| Wave I/III Fair/poor self-rated health (vs. excellent) | 1.60 (1.02, 2.52) | 1.07 (0.60, 1.88) |
| Wave I/III Good self-rated health (vs. excellent) | 1.45 (1.08, 1.96) | 1.55 (1.09, 2.19) |
| Wave I/III Very good self-rated health (vs. excellent) | 1.32 (1.00, 1.75) | 1.31 (0.98, 1.74) |
| Wave I/III Depressive symptoms | 0.89 (0.66, 1.19) | 1.20 (0.92, 1.58) |
|  | **2-3 days a month** | |
| Wave I/III PSE ≤ 50% | 0.73 (0.43, 1.21) | 1.18 (0.58, 2.38) |
| Wave I/III PSE "A good chance" | 0.60 (0.39, 0.92) | 0.64 (0.34, 1.20) |
| Age (years) | 0.87 (0.78, 0.97) | 0.85 (0.74, 0.97) |
| Male | 2.17 (1.53, 3.06) | 2.40 (1.61, 3.57) |
| Foreign-born (vs. US-born) | 0.57 (0.21, 1.54) | 0.44 (0.12, 1.56) |
| Black, non-Hispanic (vs. white, non-Hispanic) | 0.36 (0.15, 0.86) | 0.44 (0.17, 1.12) |
| Hispanic (vs. white, non-Hispanic) | 0.61 (0.32, 1.18) | 0.78 (0.40, 1.54) |
| Asian, non-Hispanic (vs. white, non-Hispanic) | 0.69 (0.21, 2.33) | 0.41 (0.08, 2.17) |
| Multiracial, non-Hispanic (vs. white, non-Hispanic) | 1.05 (0.50, 2.20) | 1.14 (0.50, 2.63) |
| Other, non-Hispanic (vs. white, non-Hispanic) | 0.70 (0.13, 3.74) | 1.30 (0.26, 6.59) |
| Parental education < high school (vs. ≥ college) | 0.93 (0.44, 1.96) | 0.85 (0.42, 1.71) |
| Parental education high school or GED (vs. ≥ college) | 0.85 (0.52, 1.38) | 0.83 (0.50, 1.39) |
| Parental education some college (vs. ≥ college) | 1.05 (0.64, 1.72) | 0.99 (0.55, 1.76) |
| Wave I/III Block group poverty rate, % | 1.00 (0.97, 1.02) | 0.99 (0.97, 1.00) |
| Family structure: Two parents (vs. two biological parents) | 1.33 (0.80, 2.22) | 1.55 (0.90, 2.70) |
| Family structure: Single parent/other (vs. two biological parents) | 1.25 (0.81, 1.94) | 1.31 (0.77, 2.23) |
| Wave I/III Parental attachment/support | 1.06 (0.82, 1.38) | 0.99 (0.90, 1.09) |
| Childhood physical maltreatment | 1.08 (0.93, 1.25) | 1.05 (0.90, 1.24) |
| Childhood sexual abuse | 1.15 (0.90, 1.48) | 1.11 (0.82, 1.51) |
| (Lack of) Religiosity | 1.24 (1.05, 1.47) | 1.78 (1.33, 2.37) |
| Wave I/III Fair/poor self-rated health (vs. excellent) | 1.48 (0.73, 2.99) | 2.45 (0.88, 6.78) |
| Wave I/III Good self-rated health (vs. excellent) | 1.15 (0.70, 1.88) | 1.88 (0.99, 3.54) |
| Wave I/III Very good self-rated health (vs. excellent) | 1.29 (0.85, 1.96) | 2.43 (1.46, 4.06) |
| Wave I/III Depressive symptoms | 2.12 (1.43, 3.14) | 1.86 (1.21, 2.88) |
|  | **≥ Weekly** | |
| Wave I/III PSE ≤ 50% | 1.68 (1.20, 2.35) | 2.17 (1.34, 3.50) |
| Wave I/III PSE "A good chance" | 1.38 (1.04, 1.82) | 0.98 (0.60, 1.60) |
| Age (years) | 0.89 (0.81, 0.97) | 0.95 (0.87, 1.04) |
| Male | 2.02 (1.53, 2.67) | 2.00 (1.45, 2.75) |
| Foreign-born (vs. US-born) | 0.33 (0.12, 0.89) | 0.38 (0.12, 1.19) |
| Black, non-Hispanic (vs. white, non-Hispanic) | 0.22 (0.12, 0.39) | 0.26 (0.14, 0.46) |
| Hispanic (vs. white, non-Hispanic) | 0.77 (0.42, 1.40) | 0.80 (0.41, 1.55) |
| Asian, non-Hispanic (vs. white, non-Hispanic) | 0.75 (0.28, 2.04) | 0.64 (0.23, 1.75) |
| Multiracial, non-Hispanic (vs. white, non-Hispanic) | 1.03 (0.65, 1.64) | 1.33 (0.71, 2.47) |
| Other, non-Hispanic (vs. white, non-Hispanic) | 0.26 (0.03, 2.15) | 0.46 (0.06, 3.30) |
| Parental education < high school (vs. ≥ college) | 1.14 (0.69, 1.87) | 1.42 (0.85, 2.37) |
| Parental education high school or GED (vs. ≥ college) | 1.11 (0.74, 1.65) | 1.23 (0.81, 1.86) |
| Parental education some college (vs. ≥ college) | 1.34 (0.93, 1.92) | 1.43 (0.93, 2.21) |
| Wave I/III Block group poverty rate, % | 1.01 (0.99, 1.02) | 1.00 (0.99, 1.02) |
| Family structure: Two parents (vs. two biological parents) | 1.16 (0.79, 1.71) | 1.05 (0.67, 1.64) |
| Family structure: Single parent/other (vs. two biological parents) | 1.17 (0.83, 1.65) | 1.10 (0.74, 1.64) |
| Wave I/III Parental attachment/support | 1.13 (0.92, 1.38) | 1.06 (0.99, 1.14) |
| Childhood physical maltreatment | 1.07 (0.99, 1.17) | 1.04 (0.94, 1.15) |
| Childhood sexual abuse | 1.15 (0.99, 1.35) | 1.09 (0.88, 1.35) |
| (Lack of) Religiosity | 1.14 (1.00, 1.31) | 1.35 (1.05, 1.74) |
| Wave I/III Fair/poor self-rated health (vs. excellent) | 1.93 (1.18, 3.15) | 2.22 (1.09, 4.52) |
| Wave I/III Good self-rated health (vs. excellent) | 1.89 (1.28, 2.79) | 1.67 (1.05, 2.66) |
| Wave I/III Very good self-rated health (vs. excellent) | 1.65 (1.18, 2.32) | 1.45 (0.96, 2.21) |
| Wave I/III Depressive symptoms | 1.65 (1.16, 2.34) | 1.65 (1.18, 2.30) |
