# Supplementary material for: Adolescent Expectations of Early Death Predict Adult Risk Behaviors
Source: PLoS One. 2012 Aug 1;7(8):e41905. doi: 10.1371/journal.pone.0041905 (PMC3411584; doi:10.1371/journal.pone.0041905)
Supplement: Table S7 — Perceived Survival Expectations (PSE) as a predictor of binge drinking at Wave IV, Add Health. (DOCX) [file pone.0041905.s007.docx]

| Table S7. Perceived Survival Expectations (PSE) as a predictor of binge drinking at Wave IV, Add Health | | |
| --- | --- | --- |
|  | **Wave I** | **Wave III** |
|  | **AOR (95% CI)^b^** | **AOR (95% CI)^b^** |
|  | **≤ Monthly** | |
| Wave I/III PSE ≤ 50% | 0.72 (0.60, 0.87) | 0.73 (0.55, 0.96) |
| Wave I/III PSE "A good chance" | 1.07 (0.95, 1.20) | 0.91 (0.78, 1.07) |
| Age (years) | 0.94 (0.91, 0.97) | 0.93 (0.89, 0.97) |
| Male | 1.37 (1.19, 1.57) | 1.39 (1.21, 1.60) |
| Foreign-born (vs. US-born) | 1.02 (0.79, 1.32) | 0.96 (0.69, 1.34) |
| Black, non-Hispanic (vs. white, non-Hispanic) | 0.42 (0.34, 0.51) | 0.41 (0.33, 0.52) |
| Hispanic (vs. white, non-Hispanic) | 0.79 (0.63, 0.99) | 0.82 (0.64, 1.05) |
| Asian, non-Hispanic (vs. white, non-Hispanic) | 0.73 (0.54, 0.98) | 0.75 (0.54, 1.06) |
| Multiracial, non-Hispanic (vs. white, non-Hispanic) | 0.79 (0.60, 1.03) | 0.81 (0.61, 1.09) |
| Other, non-Hispanic (vs. white, non-Hispanic) | 0.55 (0.34, 0.91) | 0.65 (0.38, 1.13) |
| Parental education < high school (vs. ≥ college) | 0.65 (0.50, 0.84) | 0.53 (0.41, 0.70) |
| Parental education high school or GED (vs. ≥ college) | 0.76 (0.62, 0.92) | 0.69 (0.56, 0.85) |
| Parental education some college (vs. ≥ college) | 0.91 (0.78, 1.06) | 0.85 (0.71, 1.01) |
| Wave I/III Block group poverty rate, % | 0.99 (0.98, 1.00) | 1.00 (0.99, 1.00) |
| Family structure: Two parents (vs. two biological parents) | 0.95 (0.80, 1.12) | 0.94 (0.78, 1.13) |
| Family structure: Single parent/other (vs. two biological parents) | 0.97 (0.85, 1.12) | 0.94 (0.81, 1.10) |
| Wave I/III Parental attachment/support | 0.99 (0.90, 1.08) | 1.00 (0.96, 1.03) |
| Childhood physical maltreatment | 1.08 (1.03, 1.12) | 1.08 (1.02, 1.14) |
| Childhood sexual abuse | 1.05 (0.97, 1.14) | 1.03 (0.93, 1.13) |
| (Lack of) Religiosity | 1.09 (1.03, 1.16) | 1.43 (1.30, 1.57) |
| Wave I/III Fair/poor self-rated health (vs. excellent) | 0.84 (0.65, 1.08) | 0.63 (0.45, 0.87) |
| Wave I/III Good self-rated health (vs. excellent) | 0.88 (0.76, 1.03) | 0.86 (0.73, 1.02) |
| Wave I/III Very good self-rated health (vs. excellent) | 1.13 (0.99, 1.29) | 1.07 (0.91, 1.24) |
| Wave I/III Depressive symptoms | 0.93 (0.78, 1.12) | 1.12 (0.94, 1.32) |
|  | **2-3 days a month** | |
| Wave I/III PSE ≤ 50% | 0.71 (0.53, 0.96) | 0.69 (0.45, 1.07) |
| Wave I/III PSE "A good chance" | 0.93 (0.77, 1.13) | 0.93 (0.73, 1.17) |
| Age (years) | 0.89 (0.85, 0.93) | 0.87 (0.83, 0.92) |
| Male | 2.06 (1.74, 2.45) | 1.96 (1.63, 2.36) |
| Foreign-born (vs. US-born) | 0.69 (0.43, 1.13) | 0.70 (0.41, 1.19) |
| Black, non-Hispanic (vs. white, non-Hispanic) | 0.47 (0.34, 0.65) | 0.44 (0.31, 0.64) |
| Hispanic (vs. white, non-Hispanic) | 0.90 (0.64, 1.25) | 0.98 (0.69, 1.38) |
| Asian, non-Hispanic (vs. white, non-Hispanic) | 0.75 (0.42, 1.34) | 0.64 (0.34, 1.20) |
| Multiracial, non-Hispanic (vs. white, non-Hispanic) | 0.98 (0.65, 1.48) | 0.97 (0.67, 1.41) |
| Other, non-Hispanic (vs. white, non-Hispanic) | 0.84 (0.40, 1.77) | 0.69 (0.25, 1.87) |
| Parental education < high school (vs. ≥ college) | 0.55 (0.38, 0.78) | 0.46 (0.31, 0.69) |
| Parental education high school or GED (vs. ≥ college) | 0.95 (0.73, 1.22) | 0.81 (0.63, 1.04) |
| Parental education some college (vs. ≥ college) | 0.88 (0.72, 1.06) | 0.69 (0.55, 0.86) |
| Wave I/III Block group poverty rate, % | 0.99 (0.98, 1.00) | 1.00 (1.00, 1.01) |
| Family structure: Two parents (vs. two biological parents) | 0.91 (0.73, 1.13) | 0.81 (0.62, 1.06) |
| Family structure: Single parent/other (vs. two biological parents) | 0.97 (0.77, 1.21) | 0.92 (0.72, 1.16) |
| Wave I/III Parental attachment/support | 1.01 (0.88, 1.17) | 0.98 (0.93, 1.03) |
| Childhood physical maltreatment | 1.09 (1.01, 1.18) | 1.09 (1.02, 1.18) |
| Childhood sexual abuse | 1.04 (0.92, 1.18) | 1.05 (0.90, 1.22) |
| (Lack of) Religiosity | 1.20 (1.09, 1.32) | 1.70 (1.48, 1.94) |
| Wave I/III Fair/poor self-rated health (vs. excellent) | 0.82 (0.56, 1.19) | 1.01 (0.63, 1.63) |
| Wave I/III Good self-rated health (vs. excellent) | 0.99 (0.76, 1.28) | 0.87 (0.64, 1.17) |
| Wave I/III Very good self-rated health (vs. excellent) | 1.47 (1.21, 1.79) | 1.15 (0.90, 1.47) |
| Wave I/III Depressive symptoms | 0.92 (0.70, 1.21) | 0.94 (0.72, 1.21) |
|  | **≥ Weekly** | |
| Wave I/III PSE ≤ 50% | 0.98 (0.76, 1.25) | 0.91 (0.63, 1.30) |
| Wave I/III PSE "A good chance" | 1.15 (0.93, 1.41) | 1.02 (0.80, 1.29) |
| Age (years) | 0.92 (0.87, 0.96) | 0.90 (0.86, 0.95) |
| Male | 3.48 (2.96, 4.10) | 3.22 (2.70, 3.85) |
| Foreign-born (vs. US-born) | 0.44 (0.27, 0.71) | 0.43 (0.23, 0.81) |
| Black, non-Hispanic (vs. white, non-Hispanic) | 0.44 (0.32, 0.60) | 0.38 (0.27, 0.55) |
| Hispanic (vs. white, non-Hispanic) | 0.93 (0.69, 1.25) | 0.96 (0.70, 1.30) |
| Asian, non-Hispanic (vs. white, non-Hispanic) | 0.61 (0.30, 1.22) | 0.53 (0.23, 1.23) |
| Multiracial, non-Hispanic (vs. white, non-Hispanic) | 0.89 (0.59, 1.36) | 0.97 (0.62, 1.51) |
| Other, non-Hispanic (vs. white, non-Hispanic) | 1.07 (0.50, 2.28) | 1.29 (0.61, 2.73) |
| Parental education < high school (vs. ≥ college) | 0.56 (0.40, 0.76) | 0.47 (0.32, 0.69) |
| Parental education high school or GED (vs. ≥ college) | 0.65 (0.50, 0.84) | 0.54 (0.41, 0.71) |
| Parental education some college (vs. ≥ college) | 0.86 (0.71, 1.05) | 0.77 (0.61, 0.96) |
| Wave I/III Block group poverty rate, % | 0.99 (0.98, 1.00) | 1.00 (0.99, 1.01) |
| Family structure: Two parents (vs. two biological parents) | 0.77 (0.63, 0.94) | 0.76 (0.59, 0.98) |
| Family structure: Single parent/other (vs. two biological parents) | 1.12 (0.92, 1.37) | 1.11 (0.90, 1.38) |
| Wave I/III Parental attachment/support | 1.10 (0.99, 1.23) | 1.02 (0.97, 1.07) |
| Childhood physical maltreatment | 1.03 (0.97, 1.10) | 1.02 (0.94, 1.10) |
| Childhood sexual abuse | 0.99 (0.87, 1.12) | 1.02 (0.87, 1.19) |
| (Lack of) Religiosity | 1.26 (1.15, 1.38) | 1.96 (1.68, 2.29) |
| Wave I/III Fair/poor self-rated health (vs. excellent) | 0.90 (0.64, 1.28) | 0.78 (0.50, 1.20) |
| Wave I/III Good self-rated health (vs. excellent) | 1.10 (0.86, 1.40) | 0.96 (0.75, 1.25) |
| Wave I/III Very good self-rated health (vs. excellent) | 1.01 (0.83, 1.22) | 1.19 (1.00, 1.41) |
| Wave I/III Depressive symptoms | 1.13 (0.91, 1.41) | 1.15 (0.90, 1.47) |
