# Supplementary material for: Adolescent Expectations of Early Death Predict Adult Risk Behaviors
Source: PLoS One. 2012 Aug 1;7(8):e41905. doi: 10.1371/journal.pone.0041905 (PMC3411584; doi:10.1371/journal.pone.0041905)
Supplement: Table S8 — Perceived Survival Expectations (PSE) as a predictor of marijuana use at Wave IV, Add Health. (DOCX) [file pone.0041905.s008.docx]

| Table S8. Perceived Survival Expectations (PSE) as a predictor of marijuana use at Wave IV, Add Health | | |
| --- | --- | --- |
|  | **Wave I** | **Wave III** |
|  | **AOR (95% CI)^b^** | **AOR (95% CI)^b^** |
|  | **≤ Monthly** | |
| Wave I/III PSE ≤ 50% | 0.81 (0.61, 1.07) | 1.17 (0.82, 1.67) |
| Wave I/III PSE "A good chance" | 0.98 (0.81, 1.18) | 0.82 (0.63, 1.06) |
| Age (years) | 0.88 (0.83, 0.92) | 0.87 (0.82, 0.92) |
| Male | 1.50 (1.27, 1.78) | 1.52 (1.27, 1.82) |
| Foreign-born (vs. US-born) | 0.69 (0.45, 1.05) | 0.80 (0.49, 1.28) |
| Black, non-Hispanic (vs. white, non-Hispanic) | 0.79 (0.61, 1.02) | 0.70 (0.53, 0.93) |
| Hispanic (vs. white, non-Hispanic) | 0.98 (0.71, 1.36) | 1.07 (0.77, 1.49) |
| Asian, non-Hispanic (vs. white, non-Hispanic) | 0.93 (0.55, 1.57) | 0.92 (0.54, 1.55) |
| Multiracial, non-Hispanic (vs. white, non-Hispanic) | 1.19 (0.86, 1.65) | 1.13 (0.76, 1.70) |
| Other, non-Hispanic (vs. white, non-Hispanic) | 1.14 (0.60, 2.20) | 1.37 (0.66, 2.82) |
| Parental education < high school (vs. ≥ college) | 0.50 (0.36, 0.69) | 0.34 (0.22, 0.52) |
| Parental education high school or GED (vs. ≥ college) | 0.60 (0.49, 0.75) | 0.51 (0.40, 0.65) |
| Parental education some college (vs. ≥ college) | 0.80 (0.65, 0.99) | 0.74 (0.58, 0.94) |
| Wave I/III Block group poverty rate, % | 0.99 (0.98, 1.00) | 1.00 (0.99, 1.00) |
| Family structure: Two parents (vs. two biological parents) | 1.28 (1.05, 1.55) | 1.27 (1.01, 1.60) |
| Family structure: Single parent/other (vs. two biological parents) | 1.32 (1.08, 1.62) | 1.41 (1.14, 1.75) |
| Wave I/III Parental attachment/support | 1.13 (0.97, 1.32) | 0.97 (0.92, 1.01) |
| Childhood physical maltreatment | 1.05 (0.98, 1.12) | 1.03 (0.96, 1.11) |
| Childhood sexual abuse | 1.09 (0.97, 1.23) | 1.12 (0.97, 1.30) |
| (Lack of) Religiosity | 1.18 (1.08, 1.30) | 1.58 (1.36, 1.84) |
| Wave I/III Fair/poor self-rated health (vs. excellent) | 1.24 (0.91, 1.70) | 1.02 (0.63, 1.65) |
| Wave I/III Good self-rated health (vs. excellent) | 1.22 (0.99, 1.51) | 1.20 (0.93, 1.54) |
| Wave I/III Very good self-rated health (vs. excellent) | 1.23 (1.00, 1.52) | 1.25 (1.01, 1.54) |
| Wave I/III Depressive symptoms | 1.15 (0.96, 1.39) | 1.35 (1.10, 1.65) |
|  | **2-3 days a month** | |
| Wave I/III PSE ≤ 50% | 1.04 (0.59, 1.83) | 1.09 (0.56, 2.11) |
| Wave I/III PSE "A good chance" | 1.03 (0.71, 1.48) | 1.01 (0.63, 1.61) |
| Age (years) | 0.82 (0.75, 0.90) | 0.79 (0.72, 0.87) |
| Male | 2.27 (1.66, 3.10) | 2.20 (1.56, 3.10) |
| Foreign-born (vs. US-born) | 0.40 (0.16, 0.97) | 0.56 (0.25, 1.30) |
| Black, non-Hispanic (vs. white, non-Hispanic) | 0.85 (0.53, 1.36) | 0.86 (0.48, 1.53) |
| Hispanic (vs. white, non-Hispanic) | 1.07 (0.61, 1.90) | 1.12 (0.59, 2.12) |
| Asian, non-Hispanic (vs. white, non-Hispanic) | 0.95 (0.52, 1.74) | 1.13 (0.58, 2.20) |
| Multiracial, non-Hispanic (vs. white, non-Hispanic) | 1.55 (0.88, 2.71) | 1.57 (0.78, 3.16) |
| Other, non-Hispanic (vs. white, non-Hispanic) | 0.14 (0.03, 0.69) | 0.18 (0.04, 0.92) |
| Parental education < high school (vs. ≥ college) | 0.56 (0.29, 1.08) | 0.61 (0.31, 1.20) |
| Parental education high school or GED (vs. ≥ college) | 0.42 (0.28, 0.63) | 0.48 (0.30, 0.77) |
| Parental education some college (vs. ≥ college) | 0.72 (0.49, 1.06) | 0.71 (0.48, 1.05) |
| Wave I/III Block group poverty rate, % | 1.00 (0.98, 1.01) | 1.00 (0.99, 1.01) |
| Family structure: Two parents (vs. two biological parents) | 0.79 (0.50, 1.25) | 0.93 (0.55, 1.57) |
| Family structure: Single parent/other (vs. two biological parents) | 1.07 (0.75, 1.52) | 1.09 (0.72, 1.64) |
| Wave I/III Parental attachment/support | 1.27 (1.02, 1.58) | 1.01 (0.95, 1.08) |
| Childhood physical maltreatment | 1.05 (0.91, 1.20) | 1.06 (0.92, 1.22) |
| Childhood sexual abuse | 0.99 (0.73, 1.33) | 0.92 (0.60, 1.41) |
| (Lack of) Religiosity | 1.21 (1.03, 1.41) | 1.47 (1.16, 1.87) |
| Wave I/III Fair/poor self-rated health (vs. excellent) | 1.52 (0.74, 3.12) | 2.34 (1.11, 4.91) |
| Wave I/III Good self-rated health (vs. excellent) | 2.53 (1.62, 3.95) | 2.15 (1.27, 3.64) |
| Wave I/III Very good self-rated health (vs. excellent) | 1.70 (1.10, 2.63) | 2.25 (1.47, 3.45) |
| Wave I/III Depressive symptoms | 0.96 (0.62, 1.50) | 1.21 (0.87, 1.68) |
|  | **≥ Weekly** | |
| Wave I/III PSE ≤ 50% | 1.08 (0.85, 1.37) | 1.12 (0.81, 1.57) |
| Wave I/III PSE "A good chance" | 0.83 (0.70, 1.00) | 1.02 (0.81, 1.29) |
| Age (years) | 0.88 (0.84, 0.92) | 0.90 (0.85, 0.95) |
| Male | 2.49 (2.11, 2.95) | 2.80 (2.34, 3.36) |
| Foreign-born (vs. US-born) | 0.41 (0.24, 0.70) | 0.19 (0.09, 0.40) |
| Black, non-Hispanic (vs. white, non-Hispanic) | 1.19 (0.94, 1.50) | 1.17 (0.85, 1.60) |
| Hispanic (vs. white, non-Hispanic) | 0.86 (0.62, 1.18) | 0.95 (0.68, 1.34) |
| Asian, non-Hispanic (vs. white, non-Hispanic) | 0.70 (0.42, 1.15) | 0.74 (0.43, 1.27) |
| Multiracial, non-Hispanic (vs. white, non-Hispanic) | 1.52 (1.11, 2.08) | 1.51 (1.07, 2.15) |
| Other, non-Hispanic (vs. white, non-Hispanic) | 0.83 (0.33, 2.05) | 0.71 (0.25, 1.98) |
| Parental education < high school (vs. ≥ college) | 0.77 (0.55, 1.09) | 0.62 (0.43, 0.90) |
| Parental education high school or GED (vs. ≥ college) | 0.88 (0.72, 1.09) | 0.80 (0.65, 0.99) |
| Parental education some college (vs. ≥ college) | 1.02 (0.81, 1.29) | 0.94 (0.73, 1.20) |
| Wave I/III Block group poverty rate, % | 0.99 (0.98, 1.00) | 1.00 (0.99, 1.00) |
| Family structure: Two parents (vs. two biological parents) | 1.32 (1.05, 1.66) | 1.28 (1.01, 1.63) |
| Family structure: Single parent/other (vs. two biological parents) | 1.50 (1.22, 1.85) | 1.63 (1.29, 2.06) |
| Wave I/III Parental attachment/support | 1.19 (1.04, 1.35) | 1.01 (0.96, 1.05) |
| Childhood physical maltreatment | 1.15 (1.10, 1.21) | 1.13 (1.07, 1.21) |
| Childhood sexual abuse | 1.09 (0.99, 1.20) | 1.06 (0.95, 1.20) |
| (Lack of) Religiosity | 1.30 (1.20, 1.42) | 1.83 (1.59, 2.11) |
| Wave I/III Fair/poor self-rated health (vs. excellent) | 1.55 (1.12, 2.15) | 1.32 (0.83, 2.11) |
| Wave I/III Good self-rated health (vs. excellent) | 1.42 (1.13, 1.77) | 1.59 (1.23, 2.05) |
| Wave I/III Very good self-rated health (vs. excellent) | 1.33 (1.08, 1.65) | 1.44 (1.14, 1.82) |
| Wave I/III Depressive symptoms | 1.13 (0.88, 1.45) | 1.44 (1.17, 1.77) |
